# PET–CT benchmarked detection and 5-year progression of asymptomatic tuberculosis: a longitudinal, prospective cohort study

**DOI:** 10.1016/S2213-2600(26)00056-1

**Published:** 2026-06

**Authors:** Hanif Esmail, Friedrich Thienemann, Bianca Sossen, Sandra L Mukasa, Francisco Lakay, Jacob E Munro, Liana Macpherson, James M Warwick, Rene T Goliath, Nashreen Omar-Davies, Emily Douglass, Amanda Jackson, Elizabeth M Streicher, Torben Heinsohn, Dylan Sheerin, Marilou H Barrios, Saalikha Aziz, Keboile C Serole, Remy Daroowala, Arshad Taliep, Petri Ahlers, Stephanus T Malherbe, Rory Bowden, Rob M Warren, Gerhard Walzl, Laura E Via, Melanie Bahlo, Sandra V Kik, Morten Ruhwald, Karen R Jacobson, C Robert Horsburgh, Padmini Salgame, David Alland, Clifton E Barry, JoAnne L Flynn, Jerrold J Ellner, Anna K Coussens, Robert J Wilkinson

**Affiliations:** a Wellcome Discovery Research Platforms in Infection, Centre for Infectious Diseases Research in Africa, Institute of Infectious Disease and Molecular Medicine, University of Cape Town, Cape Town, South Africa; b Department of Medicine, University of Cape Town, Cape Town, South Africa; c Department of Pathology, University of Cape Town, Cape Town, South Africa; d MRC Clinical Trials Unit, University College London, London, UK; e WHO Collaborating Centre for Tuberculosis Research and Innovation, UCL Centre for Global Tuberculosis Research, Institute for Global Health, University College London, London, UK; f Department of Internal Medicine, University Hospital of Zurich, University of Zurich, Switzerland; g The Walter and Eliza Hall Institute of Medical Research, Parkville, VIC, Australia; h Department of Medical Biology, University of Melbourne, Parkville, VIC, Australia; i Division of Nuclear Medicine, Department of Medical Imaging and Clinical Oncology, Stellenbosch University, Cape Town, South Africa; j Division of Infectious Diseases, Center for Emerging Pathogens, New Jersey Medical School, Rutgers University, Newark, NJ, USA; k Department of Science and Technology/National Research Foundation Centre of Excellence in Biomedical Tuberculosis Research, South African Medical Research Council for Tuberculosis Research, Division of Molecular Biology and Human Genetics, Department of Biomedical Sciences, Faculty of Health Sciences, Stellenbosch University, Cape Town, South Africa; l Department of Epidemiology, Helmholtz Centre for Infection Research, Braunschweig, Germany; m Tuberculosis Research Section, Laboratory of Clinical Immunology and Microbiology, National Institute of Allergy and Infectious Diseases, National Institutes of Health, Bethesda, MD, USA; n Tuberculosis Imaging Program, Division of Intramural Research, National Institute of Allergy and Infectious Diseases, National Institutes of Health, Bethesda, MD, USA; o FIND, Grand-Saconnex, Switzerland; p Novo Nordisk Foundation Initiative for Vaccines and Immunity, Copenhagen, Denmark; q Department of Epidemiology, Boston University School of Public Health Boston, MA, USA; r Section of Infectious Diseases, Boston University Chobanian and Avedisian School of Medicine and Boston Medical Center, Boston, MA, USA; s Public Health Research Institute, New Jersey Medical School, Rutgers University, Newark, NJ, USA; t Department of Microbiology and Molecular Genetics, University of Pittsburgh School of Medicine, PA, USA; u The Francis Crick Institute London, London, UK; v Department of Infectious Diseases, Imperial College London, London, UK

## Abstract

**Background:**

To understand how lung pathology relates to symptoms, microbiology, and progression risk in tuberculosis and to advance diagnostic development, we determined the frequency at screening of tuberculosis-consistent lesions in asymptomatic individuals within 5 years of tuberculosis diagnosis using highly sensitive imaging ([^18^F]-fluorodeoxyglucose PET–CT) and compared with chest x-ray computer-aided detection (CAD).

**Methods:**

We enrolled a prospective longitudinal cohort in Khayelitsha, Cape Town, South Africa, of asymptomatic, HIV-uninfected contacts aged 18–65 years of patients with rifampicin-resistant tuberculosis, a tuberculosis high-risk group not eligible for chemoprophylaxis. Participants underwent baseline PET–CT, chest x-ray, phlebotomy, and intensive sputum collection, and were classified into four PET–CT lung categories: consistent with tuberculosis, inactive tuberculosis, other lesions, and normal. Chest x-ray was processed by three types of CAD software (CAD4TB [version 7.0], qXR [version 3.0.0], and Lunit [version 3.1.4.111]). Follow-up included symptom-agnostic tuberculosis screening (23–38 months) and provincial register review (≤74 months), and a subgroup had repeat PET–CT (5–15 months). Tuberculosis was defined as bacteriologically confirmed or clinically diagnosed. The primary outcome measures were hazard ratio (HR) for tuberculosis diagnosis and treatment by baseline PET–CT lung abnormality category with normal as the reference group, and diagnostic performance of chest x-ray CAD software using area under the receiver operator characteristic curve (AUC).

**Findings:**

250 asymptomatic adults were enrolled between March 3, 2015, and Oct 11, 2017, irrespective of tuberculosis history or previous infection, and followed up for 1107 person-years (median 4·7 years [IQR 4·0–5·1]). 18 (7%) participants were treated for tuberculosis (16 [89%] of 18 bacteriologically confirmed). Six of 18 participants were diagnosed at baseline (four requiring induced sputum culture) and 12 of 18 after a median of 32 months (IQR 12–35). By baseline PET–CT category, tuberculosis was diagnosed and treated in 12 (41%) of 29 participants with scans consistent with tuberculosis, two (7%) of 30 with scans consistent with inactive tuberculosis, two (2%) of 83 with scans showing other lesions, and two (2%) of 108 with scans showing normal lungs. Participants with baseline PET–CT scans consistent with tuberculosis had the highest risk of 5-year tuberculosis diagnosis (HR 28·54 [95% CI 6·37–127·81] compared with those with scans showing normal lungs, p<0·0001), with no significant risk for scans consistent with inactive tuberculosis (3·55 [0·50–25·21], p=0·21) or other lung lesions (1·30 [0·18–9·23], p=0·79). 11 (69%) of the 16 participants with bacteriologically confirmed tuberculosis were asymptomatic at bacteriological confirmation, and ten (91%) of 11 had baseline PET–CT scans consistent with tuberculosis. Using baseline PET–CT classification as reference, the AUC for chest x-ray CAD software ranged from 0·86 (95% CI 0·72–0·99) to 0·89 (0·75–1·00) for bacteriologically confirmed tuberculosis.

**Interpretation:**

Most adult asymptomatic contacts diagnosed with tuberculosis over 5 years had baseline radiographically evident disease, not radiographically negative incipient tuberculosis. Although PET–CT is not feasible for routine screening, it provides a highly sensitive reference benchmark for diagnostic development, with chest x-ray CAD performing comparatively well.

**Funding:**

South Africa Medical Research Council, US National Institutes of Health, Gates Foundation, Wellcome, UK Research and Innovation Medical Research Council, and Walter and Eliza Hall Institute of Medical Research.

**Research in context**

**Evidence before this study**

The development of biomarkers that predict tuberculosis progression in asymptomatic individuals has been focused on detection of radiographically negative incipient tuberculosis. However, the proportion of tuberculosis diagnosed over 2–5 years that is and is not radiographically evident at baseline screening with highly sensitive PET–CT imaging is unknown. This is important because asymptomatic tuberculosis is increasingly appreciated not only to be an opportunity for treatment intervention before substantial tissue damage but might also contribute substantially to transmission. A 2022 systematic review identified 25 studies (two PET–CT, two PET–MRI, and 21 CT studies) using high-resolution imaging of the lungs in asymptomatic household contacts of tuberculosis, and 13 of these studies included adults. We conducted a literature search for publications between Oct 26, 2021, and Feb 9, 2026, to identify more recent cohorts combining “tuberculosis” AND “positron emission tomography computed tomography” with the following terms “household”, “contacts”, “asymptomatic”, “subclinical”, “preclinical”, “incipient”, “progression”, “predictive”, and “prognostic”. One further cohort was identified from the UK with 20 adult contacts (of whom eight had PET–CT abnormalities) followed up for 12 months. Studies were small (median 30 participants [IQR 13–56]) with limited follow-up without treatment, such that no conclusion was made regarding the natural history of how lesions identified related to future tuberculosis progression.

**Added value of this study**

We conducted, to the best of our knowledge, the largest study of PET–CT in 250 asymptomatic household contacts of patients with drug-resistant tuberculosis combined with intensive sputum sampling and the most comprehensive follow-up over a median of 4·7 years with no preventive therapy as per national and international guidelines. All participants with baseline lesions consistent with tuberculosis who became sputum culture-positive did so while still asymptomatic. PET–CT abnormalities were present in more than 50% participants and 24% had lung lesions consistent with tuberculosis or inactive tuberculosis. Such lesions were present at baseline in the majority (78%) of participants diagnosed with prevalent or 5-year incident disease and those with PET–CT lesions consistent with tuberculosis had a 28·5-fold increased risk of tuberculosis diagnosis. Participants with PET–CT lesions consistent with tuberculosis or inactive tuberculosis with no tuberculosis history and in whom tuberculosis was subsequently diagnosed, compared with those in whom it was not, had lower BMI, higher total white cell count, higher neutrophil count, more infiltrates, more cavities, and higher lung maximum glycolytic uptake (SUV_max_) at baseline. Benchmarked against asymptomatic PET–CT consistent with tuberculosis, computer-aided detection (CAD) software of chest x-rays had an area under the receiver operator curve of about 0·8, supporting its further evaluation and implementation in intensified screening programmes.

**Implications of all the available evidence**

Our results challenge the current concepts of the evolution of human tuberculosis disease and have implications for diagnostic, prognostic, and intervention strategies. The 2017 WHO target product profile to predict tuberculosis progression relies on identifying individuals with radiographically negative incipient tuberculosis infection who progress to symptomatic tuberculosis within 2 years. Counter to this strategy, in a setting with a high tuberculosis burden, we found that the majority of individuals with tuberculosis infection who were diagnosed with tuberculosis within 5 years had radiographically evident asymptomatic disease at baseline and those at that time induced sputum negative can take longer than 2 years to become bacteriologically confirmed, while remaining asymptomatic. PET–CT lesions consistent with tuberculosis showed dynamic evolution, but rarely self-resolved over a year. Routine spontaneous sputum testing also missed the majority of asymptomatic tuberculosis cases that were confirmed by intensive sputum induction sampling. Chest x-ray-CAD software performed well in detecting individuals with the most clinically significant lesions on PET–CT; however, further development of diagnostics and therapeutic approaches is needed to better target this population and reduce the numbers needed to treat to prevent a case of clinical disease. Such improved diagnostics and therapeutics will enable tuberculosis care and prevention strategies to drastically limit onwards transmission and potential post-tuberculosis sequelae.

## Introduction

Passive case detection, and thereby management, of symptomatic tuberculosis alone is insufficient to interrupt disease transmission.^1^ National and subnational tuberculosis prevalence surveys conducted between 2001 and 2017 indicate that about half of individuals with undiagnosed tuberculosis in the community are asymptomatic (ie, have a negative symptom screen; also referred to as subclinical tuberculosis), with disease detected using chest x-ray and bacteriologically confirmed with a spontaneous sputum sample.^2^ In 2024, WHO provided definitions for asymptomatic tuberculosis for programmatic use, and symptom-agnostic, community-wide screening is being scaled up in many high-priority countries to identify undiagnosed cases and interrupt transmission.^3^, ^4^, ^5^ 38% of notified tuberculosis cases globally are bacteriologically unconfirmed: even in high-income settings such as the UK, this proportion is similar.^3^, ^6^ This finding highlights the limitation in the sensitivity of current sampling and microbiological approaches.

Bacteriologically unconfirmed tuberculosis is commonly evident through imaging. In a meta-analysis of 24 studies with a combined 139 212 participants from 34 longitudinal cohorts from the pre-chemotherapy era, we found that individuals with a chest x-ray suggestive of tuberculosis but who were bacteriologically negative on a sputum test were at high risk of progression to bacteriologically confirmed tuberculosis, with a 10% annualised rate over the subsequent 3 years.^7^, ^8^ Microbiological prevalence surveys also identify people with bacteriologically confirmed tuberculosis who have a chest x-ray considered to be normal, suggesting a deficit in chest x-ray sensitivity.^2^ The current target product profile for a test of tuberculosis progression in individuals with tuberculosis infection focuses on identification of radiographically negative incipient tuberculosis.^9^ However, the comparative contribution of radiographically positive asymptomatic disease to a future tuberculosis diagnosis is unknown.

Cross-sectional imaging approaches such as [^18^F]fluorodeoxyglucose ([F^18^]FDG) PET–CT have higher sensitivity to detect lung pathology than chest x-rays. In a previous study of 35 asymptomatic, HIV-infected, bacteriologically negative individuals with no tuberculosis history considered to have tuberculosis infection, all four individuals who progressed to tuberculosis disease despite isoniazid preventive therapy had baseline lesions consistent with tuberculosis detected by PET–CT.^10^ However, no large-scale study has been conducted in HIV-1 uninfected individuals. In 2021, WHO recommended computer-aided detection (CAD) software for chest x-ray interpretation,^11^ and it is being widely introduced for active case finding. CAD scores chest x-rays, and individuals with a score above a threshold considered to be suggestive of tuberculosis (which can be locally adapted) are triaged to provide sputum. The diagnostic performance of the best-performing CAD products now exceeds that of radiologists, but their performance against sensitive cross-sectional imaging has not been evaluated.^12^

We therefore aimed to determine the contribution of baseline PET–CT lesions consistent with tuberculosis to tuberculosis diagnosis during a median of 5 years of follow-up in asymptomatic HIV-uninfected people living in a high-burden tuberculosis community who were recent household contacts of patients with rifampicin-resistant tuberculosis. We also evaluated baseline chest x-ray CAD performance to detect PET–CT-evident tuberculosis and, in a subset of participants, longitudinal lesion progression in relation to tuberculosis.

## Methods

### Study design and participants

In this prospective, observational, cohort study, participants were recruited from Khayelitsha, a peri-urban township of Cape Town, South Africa in Cape Town, South Africa. Because the community tuberculosis case notification rate at study initiation was higher than 900 per 100 000 population, previous exposure to drug-sensitive tuberculosis was also possible and not excluded.

We included household contacts aged 18–65 years of patients (aged ≥15 years) with at least rifampicin-resistant tuberculosis confirmed by Xpert MTB/RIF (Cepheid, Sunnyvale, CA, USA) or culture (index cases). Household contacts were defined as individuals sleeping in the same dwelling or room, or providing themselves jointly with food or other essentials for living during the day with the person with tuberculosis for at least 7 days (continuous or non-continuous) during the 3 months before the person was diagnosed with tuberculosis. This population was chosen to enrich for a high-risk group within a high-risk setting due to their known recent exposure to rifampicin-resistant tuberculosis, for which at the time of study initiation no evidence-based therapy could be offered. Consistent with national and international guidelines at the time, adult household contacts of patients with rifampicin-resistant tuberculosis were not provided with preventive therapy.^13^, ^14^, ^15^ This treatment guideline provided the opportunity to ethically longitudinally monitor asymptomatic disease evolution in these individuals in the absence of treatment.

Household contacts consented to initial screening (appendix pp 3–4). Household contacts further consented to baseline PET–CT and chest x-ray imaging and blood and sputum sampling in the absence of the following exclusion criteria: HIV infection, symptoms of tuberculosis by 2014 South African National Guidelines (unexplained cough for ≥2 weeks, fever, weight loss, and night sweats),^14^ clinical signs of tuberculosis, acute illness, older than 65 years, smoker with more than 30 pack-years, malignancy, chronic lung infection or inflammation, inhaled or systemic steroid use within previous 2 weeks, breastfeeding, pregnant, planning pregnancy, unable to be followed up, uncontrolled diabetes, or unwilling (appendix p 3).

The study was approved by the human research ethics committees of the University of Cape Town (HREC 449/2014), Boston University (Boston, MA, USA; H-35831), Rutgers University (New Brunswick, NJ, USA; Pro2018001966), and the Division of Microbiology and Infectious Diseases of the US National Institutes of Health (Bethesda, MD, USA; DMID 16-0112). This report follows STROBE guidelines for cohort studies. Results relating to the wider cohort have been published.^16^ This report is of the asymptomatic subgroup who underwent PET–CT. All participants provided written informed consent before participation.

### Procedures

At baseline, all participants underwent tuberculosis symptom screening and posterior–anterior digital chest x-ray with the Phillips Essenta Digital Radiography (Hamburg, Germany) system. Blood sampling was performed for full blood count, C-reactive protein, erythrocyte sedimentation rate, QuantiFERON-TB (QFT; QIAGEN, Valencia, CA, USA) Gold in tube, and QFT Gold Plus (QFT-Plus; containing two different *Mycobacterium tuberculosis* antigen tubes Plus 1 and Plus 2; appendix pp 3–4). All participants were invited to produce sputum spontaneously and then underwent further sputum induction using hypertonic saline until three sputum samples were obtained, where possible. All sputum samples underwent smear, Xpert MTB/RIF, and bacterial culture (BACTEC Mycobacteria Growth Indicator Tube [MGIT]; BD Diagnostic Systems, Sparks, MD, USA), with positive cultures analysed by whole-genome sequencing and extended drug sensitivity testing (appendix pp 7–8). Following further consent, a subset of participants had repeat PET–CT imaging, blood tests, and a single induced sputum for smear, Xpert MTB/RIF, and culture performed between 5 months and 15 months, and some participants underwent bronchoalveolar lavage after repeat PET–CT for MGIT culture (appendix pp 6, 8).

PET–CT scans were performed at three different sites close to Khayelitsha using similar imaging protocols. PET–CT and chest x-ray reading is detailed in the appendix (pp 4–6). Three types of CAD software were evaluated: CAD4TB (version 7.0, Delft Imaging), qXR (version 3.0.0), and Lunit INSIGHT chest x-ray (version 3.1.4.111). CAD output consists of a continuous score (0–100 for CAD4TB and Lunit, and 0–1 for qXR), with commonly used (CAD4TB)^12^ or manufacturer-recommended (Lunit and qXR) thresholds set (50, 15, and 0·5, respectively) to identify individuals with abnormalities suggestive of tuberculosis warranting sputum investigation.

Active follow-up of all participants occurred with a clinical assessment for signs and symptoms of tuberculosis at about 12 months (range 9–16 months), with sputum investigations conducted if individuals were symptomatic and an active screening for tuberculosis irrespective of symptoms between 23 months and 38 months with three sputum samples (induced if needed) and any time before this visit if they reported tuberculosis symptoms during phone calls every 3 months from enrolment. In addition, all participants consented to passive follow-up by inspection of their health service encounters via the Western Cape Provincial Health Data Centre database, which captures illness episodes prospectively on a population-wide basis, and clinic medical records to ensure that all episodes of tuberculosis that were diagnosed or treated within the Western Cape Province were captured. The Provincial Health Data Centre database was cross-checked for additional cases on May 21, 2021, to capture all tuberculosis episodes from the start of the study to this date and to confirm previous episodes before study enrolment.^17^

### Outcomes

Baseline PET–CT scans were classified into four prespecified (appendix pp 5–6) mutually exclusive lung categories: radiographically consistent with tuberculosis disease, defined as the presence of infiltrates or FDG-avid nodules within the upper lobes or apical segment of lower lobes; radiographically consistent with inactive tuberculosis, defined as the presence of fibrotic scars within the upper lobes or apical segment of lower lobes; other parenchymal abnormalities not meeting the definitions of consistent with tuberculosis or inactive tuberculosis; and normal lung parenchyma.

The imaging reference standards to evaluate the CAD chest x-ray were all participants with baseline PET–CT scans consistent with tuberculosis and all those with baseline PET–CT scans consistent with tuberculosis or inactive tuberculosis who were bacteriologically confirmed at any point during follow-up.

For the primary analysis, participants were considered to have tuberculosis if a decision to start treatment for disease was made by a statutory provider and, within those treated, as bacteriologically confirmed tuberculosis if any specimen was positive for *M tuberculosis* by culture or PCR.

### Statistical analysis

Sample size was governed by the expected proportion of participants with PET–CT parenchymal abnormalities consistent with tuberculosis or inactive tuberculosis. We predicted 50% of participants would become infected following exposure, of whom 30–40% would have abnormalities based on previous experience.^10^ We therefore expected 15–20% of participants to have these PET–CT abnormalities, with a sample of 250 providing precision of approximately ±5% with 95% confidence.

Continuous variables were assessed for normality using Shapiro–Wilk test and consistently violated normality. Consequently, comparisons were performed using Mann–Whitney *U* test or Kruskal–Wallis with Dunn's multiple comparison test using Bonferroni adjustment. Proportions were compared by χ^2^ or Fisher's exact test. Correlation was assessed by Pearson's correlation. Receiver operating characteristic (ROC) analyses were performed and area under the ROC curve (AUC) values compared using roccomp (Stata), which tests for the equality of the AUC using DeLong's method. Kaplan–Meier failure curves, stratified by baseline PET–CT, chest x-ray CAD, and QFT status were estimated. Cox proportional hazards regression was performed to estimate hazard ratios (HRs) and 95% CIs for factors associated with a tuberculosis diagnosis. Time zero was defined as date of first PET–CT scan, and participants were censored at the earliest of study end, loss to follow-up (last date known to be tuberculosis free), or non-tuberculosis-related death. Univariate analyses were first performed for PET–CT lung category, age, sex, previous tuberculosis, QFT-positive status, and longer than 6 h of daily index case exposure. Variables included in the post-hoc multivariate model were selected on the basis of univariate significance, clinical relevance, and previous evidence. The proportional hazards assumption was assessed using Schoenfeld residuals and graphically, and was not violated. Model fit was evaluated using likelihood ratio tests. Sensitivity analyses were conducted restricting to culture-positive cases only, expanding to include those with positive microbiology irrespective of treatment, or, in exploratory post-hoc analysis, restricted to baseline QFT-positive (positive on QFT-Gold, QFT-Plus, or both) individuals with treated tuberculosis.

Statistical analysis was conducted in Stata (version 18.5) and Prism (version 10.4.1).

### Role of the funding source

The funders of the study had no role in study design, data collection, data analysis, data interpretation, writing of the report, or the decision to submit for publication.

## Results

**Figure 1:**
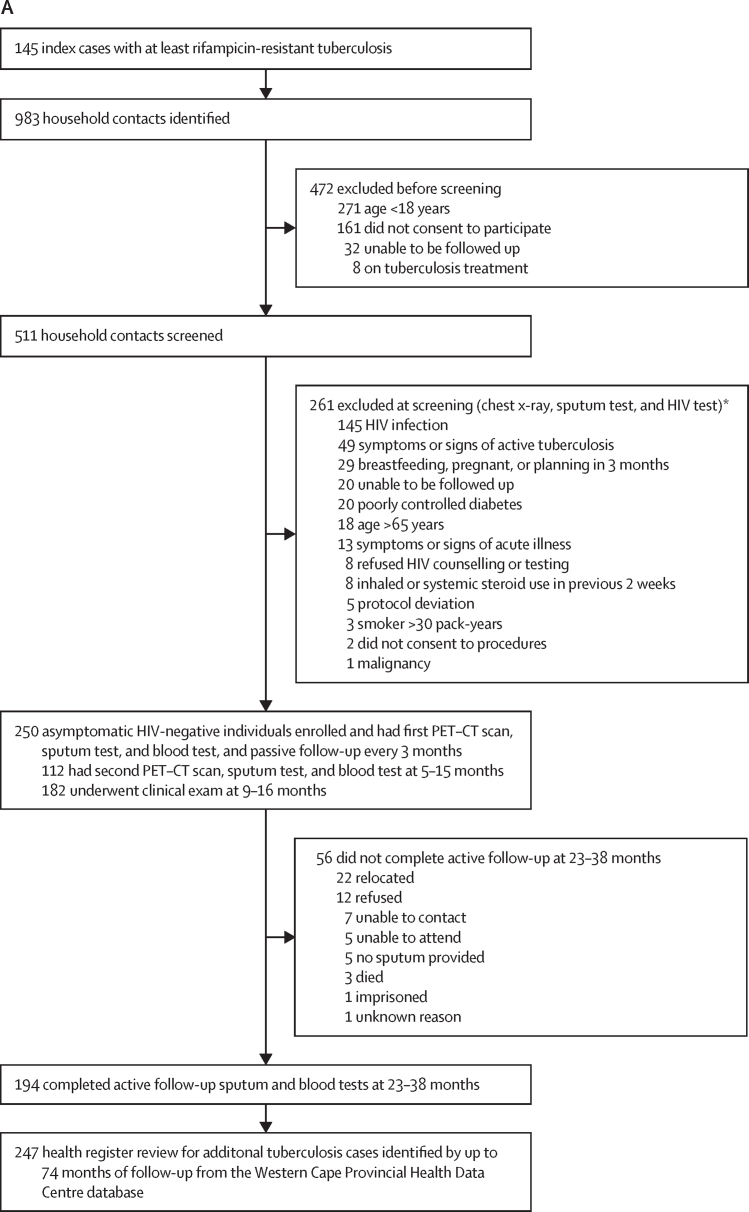

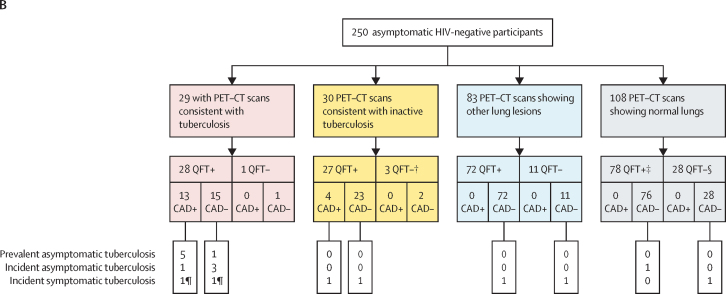
Study flowchart (A) Participant flow. (B) Baseline PET–CT lung classification by QFT (QFT-Gold or QFT-Plus; positive or negative for tuberculosis) and chest x-ray CAD4TB (positive or negative for tuberculosis) result. Tuberculosis diagnostic results for participants treated for tuberculosis at baseline or follow-up are stratified by baseline characteristics, including two with a clinical diagnosis. CAD=computer-aided detection. QFT=QuantiFERON-TB. *Individuals could be excluded for more than one reason. †One QFT-negative participant with a PET–CT scan consistent with inactive tuberculosis had no chest x-ray. ‡Two QFT-positive participants with normal lungs on PET–CT did not have valid CAD4TB reading. §Two additional participants with PET–CT scans showing normal lungs did not have baseline QFT but were CAD-negative. ¶Clinical diagnosis of tuberculosis.

Between March 3, 2015, and Oct 11, 2017, we identified 983 household contacts of 145 index cases with an *M tuberculosis* isolate resistant to at least rifampicin. 271 (28%) of 983 household contacts were younger than 18 years. Following consent, 511 adult participants were screened for eligibility, and 261 (51%) were excluded, including 145 (28%) with HIV infection and 49 (10%) whose tuberculosis symptom screen was positive. 250 HIV-negative, asymptomatic adults were enrolled, irrespective of QFT tuberculosis infection status and previous tuberculosis (figure 1).

By comparison with excluded adults, those enrolled for PET–CT were more commonly male (100 [40%] of 250 *vs* 79 [30%] of 261, p=0·026), slightly younger (median age 30 years [IQR 23–43] *vs* 35 years [28–49], p<0·0001), and significantly less likely to have had previous tuberculosis (34 [14%] *vs* 78 [31%], p<0·0001, primarily due to exclusion of those with HIV; appendix p 10). Of the 34 participants enrolled with previous tuberculosis, the last treatment ended a median of 9 years (IQR 5–20) before enrolment (table). *M tuberculosis* sensitisation (QFT-positive) was present in 205 (83%) of 248 participants based on a valid QFT-Plus result (241 participants) or QFT-Gold result (248 participants). Digital chest x-ray was performed in 249 participants.

**Table:** Baseline characteristics, clinical, and radiographic findings, and tuberculosis outcomes in asymptomatic HIV-negative household contacts of individuals with at least rifampicin-resistant tuberculosis

		**All household contacts (n=250)**	**PET–CT scan consistent with tuberculosis (n=29)**	**PET–CT scan consistent with inactive TB (n=30)**	**PET–CT scan showing other lung lesions (n=83)**	**PET–CT scan showing normal lung (n=108)**	**p value**
**Demographics**							
Age, years		30 (23–43)	43 (30–50)*†	41 (28–54)*†	28 (23–39)	27 (22–40)	<0·0001
Sex		..	..	..	..	..	0·49
	Female	150 (60%)	14 (48%)	17 (57%)	53 (64%)	66 (61%)	..
	Male	100 (40%)	15 (52%)	13 (43%)	30 (36 %)	42 (39 %)	..
Previous tuberculosis		34 (14%)	14 (48%)	15 (50%)	2 (2%)	3 (3%)	<0·0001
Time since previous tuberculosis treatment ended, years		9 (5–20)	9 (5–21)	8 (4–17)	19 (18–20)	20 (5–39)	0·49
Daily index case contact‡		208 (83%)	24 (83%)	26 (87%)	72 (87%)	86 (80%)	0·57
Contact of >6 h per day with index case		96/249 (39%)	14 (48%)	10 (33%)	37 (45%)	35/107 (33%)	0·23
Time co-habiting with index case, years		0·46 (0–10·0)	3·0 (0–12·5)	0·25 (0–10·3)	0·75 (0–10·0)	0·38 (0–5·8)	0·22
First-degree relative of index		106 (42%)	9 (31%)	19 (63%)	39 (47%)	39 (36%)	0·11
Ever smoked		85 (34%)	12 (41%)	15 (50%)	22 (27%)	36 (33%)	0·10
BMI		28·2 (22·2–34)	24·0 (19·8–29·3)*	25·5 (21·3–34·6)	29·2 (22·4–33·5)	28·5 (23·5–35·4)	0·021
**Laboratory investigations**							
QFT-Gold-positive or QFT-Plus-positive		205/248 (83%)	28 (97%)	27 (90%)	72 (87%)	78/106 (74%)	0·0071
QFT nil IFNγ, IU/mL		0·03 (0·00–0·13)	0·08 (0·01–0·14)	0·01 (0·00–0·08)	0·03 (0·00–0·18)	0·03 (0·00–0·12)	0·17
QFT-Gold antigen minus nil IFNγ, IU/mL		6·8 (0·7–41·8)	14·6 (3·1–56·9)*	10·2 (1·4–57·0)	7·6 (0·9–48·0)	3·79 (0·1–19·1)	0·012
QFT-Plus 1 antigen minus nil IFNγ, IU/mL		5·53 (0·45–29·57)	12·90 (2·39–36·93)*	10·70 (1·95–34·69)	5·53 (0·95–40·26)	2·78 (0·15–16·57)	0·018
QFT-Plus 2 antigen minus nil IFNγ, IU/mL		6·26 (0·59–33·37)	18·01 (2·58–44·46)*	10·76 (1·80–33·72)	6·49 (0·76–48·44)	3·48 (0·24–20·50)	0·018
C-reactive protein, mg/L		3 (1–7)	4 (1–11)	3·5 (1–7)	2·3 (1–5)	2 (1–7)	0·44
C-reactive protein ≥10 mg/L		40/247 (16%)	8 (28%)	4 (13%)	11/82 (13%)	17/106 (16%)	0·33
Erythrocyte sedimentation rate, mm/h		15 (5–30)	20 (8–33)	20·5 (9–31)	11 (4–30)	13·5 (3–26)	0·098
White cell count, × 10^9^ cells per L)		5·91 (4·88–7·54)	7·04 (5·97–8·01)	5·75 (5·06–7·18)	5·78 (4·54–7·39)	5·85 (4·74–7·62)	0·062
Neutrophil count, × 10^9^ cells per L		3·28 (2·27–4·54)	4·00 (3·39–5·14)*†	3·25 (2·33–4·46)	2·94 (2·18–4·64)	3·19 (2·25–4·42)	0·041
Neutrophil-to-lymphocyte ratio in blood		1·59 (1·12–2·24)	2·21 (1·60–2·67)*†	1·56 (1·21–2·32)	1·50 (1·10–2·22)	1·60 (1·07–2·09)	0·028
Neutrophil-to-monocyte ratio in blood		8·04 (6·21–10·53)	9·17 (7·13–12·24)	7·24 (5·81–10·47)	8·15 (6·49–10·40)	7·99 (6·11–10·16)	0·25
Lymphocyte-to-monocyte ratio		5·09 (4·00–6·49)	4·50 (3·47–5·85)	5·31 (3·44–6·65)	4·99 (4·31–6·77)	5·15 (4·11–6·26)	0·24
**Chest x-ray findings**							
Suggestive of tuberculosis (medical officer read)		37/249 (15%)	16 (55%)	10/29 (34%)	2 (2%)	9 (8%)	<0·0001
CAD4TB score		6·6 (3·4–19·1)	41·7 (12·7–65·0) *†	13·2 (5·2–33·0)*†	6·2 (3·0–14·5)	5·0 (3·2–9·9)	<0·0001
qXR score		0·01 (0·01–0·02)	0·29 (0·01–0·73) *†	0·03 (0·01–0·37)*†	0·01 (0·01–0·01)	0·01 (0·01–0·01)	<0·0001
Lunit score		1·3 (0·8–2·5)	54·4 (2·0–90·7)*†	4·4 (1·8–46·6)*†	1·1 (0·8–1·8)	1·0 (0·8–1·7)	<0·0001
**PET–CT imaging findings**							
Total lung lesion number		1 (0–3)	6 (4–10)*†	5 (2–8)*†	1 (1–3)*	0 (0–0)	<0·0001
	Total infiltrates	0 (0–0)	1 (0–2)†§	0 (0–0)	0 (0–0)	NA	<0·0001
	Total fibrotic scars	0 (0–2)	1 (0–2·5)†§	2 (1–3)†	0 (0–0)	NA	<0·0001
	Total nodules	1 (0·75–3)	3 (1–5)†	1 (0–4·25)	1 (1–2)	NA	0·034
	Total cavities	0 (0–0)	0 (0–0·5)†§	0 (0–0)	0 (0–0)	NA	<0·0001
At least one cavity present		8 (3%)	7 (24%)	1 (3%)	0	0	<0·0001
Largest lung lesion size, mm		11·15 (4·82–32·55)	44·19 (26·2–56·3)†	33·3 (26·5–45·7)†	5 (4–9·66)	NA	<0·0001
FDG-avid lung lesion present		54 (22%)	28 (97%)	13 (43%)	13 (16%)	0	<0·0001
Lung maximum visual score		0 (0–1)	3 (2–3)†§	0 (0–1)	0 (0–0)	NA	<0·0001
Lung SUV_max_		1·29 (0·94–1·89)	3·08 (1·77–5·94)†§	1·45 (1·18–1·9)†	1·07 (0·81–1·35)	NA	<0·0001
Lung HU_max_		115 (−43–481)	185 (88–648)†	670·5 (140–1123)†	7·5 (−129–179)	NA	<0·0001
Any lymph node lesion present		106 (42%)	20 (70%)	22 (73%)	34 (41%)	30 (28%)	<0·0001
Lymph node total lesion number		0 (0–2)	2 (0–3)*†	1·5 (0–3)*†	0 (0–2)	0 (0–1)	<0·0001
FDG-avid lymph node present		53 (21%)	16 (55%)	12 (40%)	15 (18%)	10 (9%)	<0·0001
Largest abnormal lymph node, mm		8·5 (5·8–11·2)	11·0 (8·35–13·4)	8·0 (5·42–12·2)	8·4 (5·0–10·3)	8·0 (5·4–10·0)	0·053
Lymph node maximum visual score		1·5 (1–3)	3 (2–3)*†	2 (1–3)*	1 (1–2)	1 (1–2)	0·0073
Lymph node SUV_max_		2·63 (1·90–4·10)	3·51 (2·63–4·87)*	2·60 (2·19–3·53)	2·25 (1·60–3·36)	2·11 (1·74–3·46)	0·032
Lymph node HU_max_		550 (135–1042)	275 (95–7685)	786 (158–1092)	488 (136–1013)	658 (208–1115)	0·25
**Tuberculosis outcome**							
Spontaneous sputum Xpert MTB/RIF-positive at baseline (treated)		1 (<1%)	1 (3%)	0	0	0	0·12
Bacteriologically confirmed at baseline		6 (2%)	6 (21%)	0	0	0	<0·0001
Bacteriologically confirmed at follow-up		10 (4%)	4 (14%)	2 (7%)	2 (2%)	2 (2%)	0·027
Clinically diagnosed¶ at follow-up		2 (1%)	2 (7%)	0	0	0	0·013
Xpert MTB/RIF-positive only, not treated		4 (2%)	1 (4%)	2 (7%)	0	1 (1%)	0·042
Any treated tuberculosis		18 (7%)	12 (41%)	2 (7%)	2 (2%)	2 (2%)	<0·0001
Any treated tuberculosis or Xpert MTB/RIF-positive only		22 (9%)	13 (45%)	4 (13%)	2 (2%)	3 (3%)	<0·0001
Asymptomatic at diagnosis, bacteriologically confirmed tuberculosis		11/16 (69%)	10/10 (100%)	0/2	0/2	1/2 (50%)	0·0014
Asymptomatic at diagnosis, culture-positive tuberculosis		11/14 (79%)	10/10 (100%)	0/1	0/1	1/2 (50%)	0·011

Data are n (%) or median (IQR). Index cases are individuals with at least rifampicin-resistant tuberculosis. The association between PET–CT categories and baseline characteristics was analysed for categorical variables by the χ^2^ or Fisher's exact test, and numerical variables by Kruskal–Wallis with post-hoc analysis using Dunn's multiple comparisons test. FDG=fluorodeoxyglucose. HU_max_=maximum Hounsfield units. QFT=QuantiFERON-TB. NA=not applicable. SUV_max_=maximum standardised uptake value.

* Significant in comparison with normal lung.

† Significant in comparison with other lung lesions.

‡ Contact with the index case every day for the past 3 months.

§ Significant in comparison with inactive tuberculosis.

¶ Clinically diagnosed tuberculosis was defined as symptom positive, bacteriologically unconfirmed treated tuberculosis.

According to baseline PET–CT scans, 108 (43%) of 250 participants had normal lung parenchyma and 142 (57%) had lung parenchyma abnormalities categorised by prespecified definition as consistent with tuberculosis (29 [12%]), consistent with inactive tuberculosis (30 [12%]), and other lung lesions (83 [33%]; table). Mediastinal and hilar lymph node abnormalities were detected in 106 (42%) participants, most frequently in those with PET–CT scans consistent with tuberculosis or inactive tuberculosis (p<0·0001; table; appendix pp 8, 25).

Compared with participants with normal lungs or other lung lesions on PET–CT, those with scans consistent with inactive tuberculosis or tuberculosis were significantly older and more frequently QFT positive. Participants with PET–CT scans consistent with tuberculosis also had significantly lower BMI, and higher QFT antigen-stimulated IFNγ than those with normal lungs, and higher neutrophil count and neutrophil-to-lymphocyte ratio than those with other lesions and normal lungs (table; appendix p 18).

About half of participants with PET–CT scans consistent with tuberculosis (14 [48%] of 29 participants) or inactive tuberculosis (15 [50%] of 30) had previous tuberculosis compared with two (2%) of 83 with other lung lesions and three (3%) of 108 with normal lungs on PET–CT (p<0·0001). The median years since end of previous tuberculosis treatment was 9 (5–21) for participants with PET–CT scans consistent with tuberculosis and 8 (4–17) for those with scans consistent with inactive tuberculosis, respectively (table). Participant differences by PET–CT lung categories in those without previous tuberculosis are in the appendix (p 12).

To investigate bacteriological confirmation, intensive sputum induction investigation was conducted at baseline, at any time a participant reported symptoms during active follow-up, and at the end of active follow-up, irrespective of symptoms. At baseline, 160 (64%) of 250 participants produced a sputum sample spontaneously and 249 (>99%) following induction (figure 1). Active follow-up was completed by 194 (78%) participants between 23 months and 38 months. Provincial medical encounters of 247 (99%) were tracked through medical records for tuberculosis outcomes, for a total follow-up time of 1107 person-years (median 4·7 years [IQR 4·0–5·1]).

**Figure 2:**
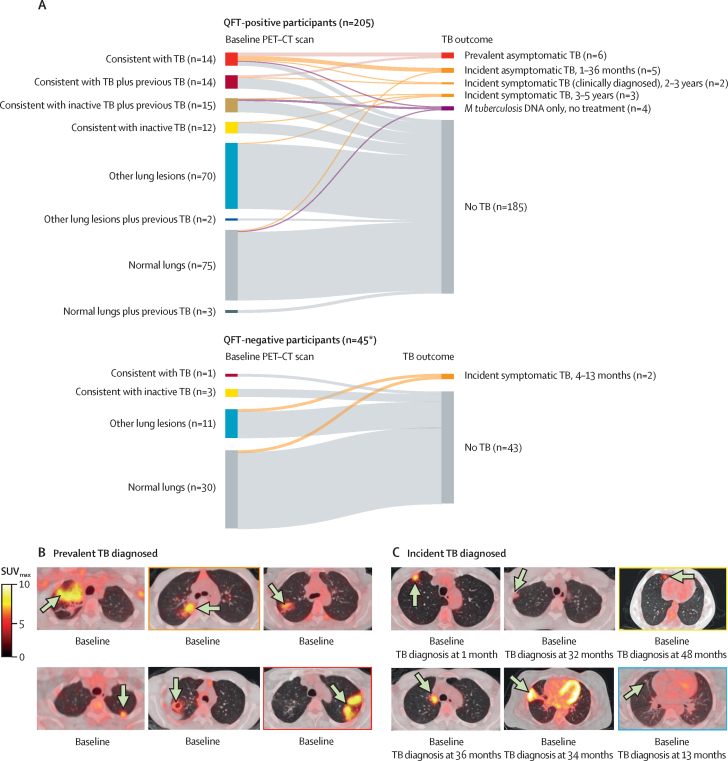
Baseline PET–CT lung abnormality category and prevalent and incident tuberculosis diagnosis outcomes of asymptomatic tuberculosis contacts (A) Sankey diagrams of the 250 asymptomatic, HIV-uninfected household contacts of patients with at least rifampicin-resistant tuberculosis according to QFT (QFT-Gold or QFT-Plus) status. (B) Example baseline axial sections of fused PET–CT of the six participants who were induced sputum culture-positive at baseline and had lesions (arrows) consistent with tuberculosis. The red box indicates the one participant who was also spontaneous sputum Xpert MTB/RIF-positive, and the orange box indicates the one participant who was also spontaneous sputum culture-positive. (C) Example baseline axial sections of fused PET–CT of six participants with no previous tuberculosis who were sputum culture-negative at baseline and diagnosed and treated for tuberculosis over follow-up. The four left images are of participants who had lesions (arrows) consistent with tuberculosis, the yellow box indicates a participant with PET–CT-inactive tuberculosis (arrow indicates infiltrate in the right middle lobe, by prespecified definition, not tuberculosis), and the blue box indicates a QFT-negative individual with other lung lesions (arrow indicates a nodule with FDG uptake not above background). FDG=fluorodeoxyglucose. *M tuberculosis=Mycobacterium tuberculosis*. QFT=QuantiFERON-TB. SUV_max_=maximum standard uptake value. TB=tuberculosis. *Includes two participants without QFT results who had baseline PET–CT scans showing normal lungs and who had no tuberculosis diagnosis.

In total, 18 (7%) of 250 participants were diagnosed with and treated for tuberculosis (16 [89%] of 18 bacteriologically confirmed [14 culture-confirmed and two Xpert MTB/RIF-positive only] and two [11%] clinically diagnosed with progressive symptoms; figure 2A). Six (33%; all bacteriologically confirmed) of 18 participants were diagnosed at baseline and 12 (67%; ten bacteriologically confirmed and two clinically diagnosed) during subsequent follow-up after a median of 32 months (IQR 12–35). At baseline, 16 (89%) participants were QFT-positive (Figure 1, Figure 2). A further four (2%) participants had a positive Xpert MTB/RIF but were culture negative (two baseline and two follow-up, all QFT-positive), and, following referral, the local clinic elected no treatment without sequelae (figure 2A). Individual radiology and microbiology for these 22 participants are detailed in the appendix (pp 13, 19–22).

Of the six participants who had bacteriologically confirmed tuberculosis at baseline, one was spontaneous sputum Xpert MTB/RIF-positive, one was spontaneous sputum culture positive, and the other four required multiple induced sputum cultures for confirmation. Of the 12 participants diagnosed and treated during follow-up, nine were identified during active follow-up (five still asymptomatic and four symptomatic) and three through medical records (symptomatic).

Tuberculosis was diagnosed and treated in 12 (41%) of 29 with baseline PET–CT scans consistent with tuberculosis (all six baseline and six [50%] of 12 follow-up cases), two (7%) of 30 with scans consistent with inactive tuberculosis, two (2%) of 83 with scans showing other lung lesions, and two (2%) of 108 with scans showing normal lungs (figure 2A–C). Of 18 participants treated for tuberculosis over the median 4·7 years, 14 (78%) had baseline PET–CT scans consistent with tuberculosis or inactive tuberculosis. Of the remaining four who did not have scans consistent with tuberculosis or inactive tuberculosis, two were QFT-negative at baseline (also with no FDG-avid lymph nodes) with symptomatic tuberculosis diagnosed after 5 months (drug-sensitive tuberculosis) and 13 months (drug-resistant tuberculosis; figure 2A, C; appendix pp 20–21). Among the 16 participants diagnosed with bacteriologically confirmed tuberculosis, 11 (69%) were asymptomatic at diagnosis and those with baseline PET–CT scans consistent with tuberculosis were significantly more likely to be asymptomatic at diagnosis (ten [100%] of ten bacteriologically confirmed) than those with scans consistent with inactive tuberculosis, showing other lesions, or showing normal lungs (one [17%] of six; p=0·0014; table; Figure 1, Figure 2).

Given the high community tuberculosis prevalence, it was not expected that all radiologically evident cases at baseline would be linked to the index case, which merely represented a recent known contact. Accordingly, of the 16 bacteriologically confirmed cases, eight (50%) were drug-resistant tuberculosis: four of six at baseline and four of ten at follow-up (appendix pp 9, 13, 19–22). Of the eight (3%) of 250 household contacts diagnosed with drug-resistant tuberculosis, seven (88%) had whole-genome sequence (if available) or drug sensitivity testing similar to the index case; six (86%) of these seven had baseline PET–CT scans consistent with tuberculosis and one (14%) was QFT-negative with a scan showing other lung lesions (appendix pp 9, 13, 19–22).

**Figure 3:**
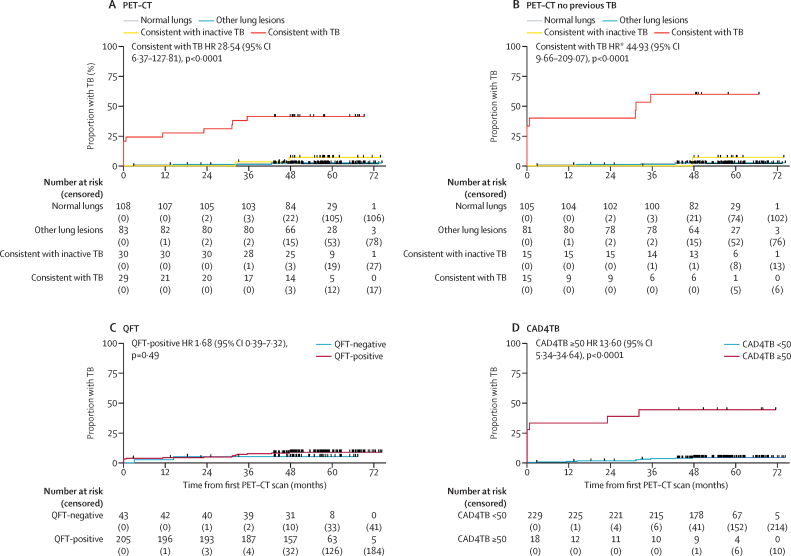
Cumulative proportion of asymptomatic household contacts diagnosed with and treated for tuberculosis at baseline and over the follow-up period according to baseline PET–CT (A, B), QFT status (C), and chest x-ray CAD (D) The event was defined as the first confirmed diagnosis of tuberculosis, based on either date of sample that resulted in positive microbiology that led to treatment or day of treatment initiated if microbiologically negative. Tick marks indicate censored participants at the earliest of end of study follow-up, loss to follow-up (last date known tuberculosis free), or non-tuberculosis death. CAD=computer-aided detection. HR=hazard ratio. TB=tuberculosis. *HR for being diagnosed and treated for tuberculosis over the study period and after adjustment for previous tuberculosis.

In the univariate analysis, risk of tuberculosis diagnosis was not associated with age, sex, previous tuberculosis, QFT-positive status, or hours of daily index case exposure (appendix pp 14–15). However, compared with participants with normal lung parenchyma on PET–CT at baseline, those with scans consistent with tuberculosis were significantly associated with an increased risk of being diagnosed and treated for tuberculosis over the median 4·7 year of follow-up (hazard ratio [HR] 28·54 [95% CI 6·37–127·81], p<0·0001), whereas there was no significant risk for those with scans consistent with inactive tuberculosis (3·55 [0·50–25·21], p=0·21) or other lung lesions (1·30 [0·18–9·23], p=0·79). In the post-hoc multivariate analysis adjusting for previous tuberculosis (a potential confounder of PET–CT lesion appearance), the adjusted HR increased to 44·93 (95% CI 9·66–209·07, p<0·0001; figure 3A, B; appendix pp 14–15). This model provided significantly better fit than the univariate model (likelihood ratio test p=0·045). Adding age and sex to this model did not further improve fit (likelihood ratio test p=0·32; appendix pp 14–15).

By contrast, QFT-positive status had a HR of 1·68 (95% CI 0·39–7·32, p=0·49; figure 3C) in the univariate analysis. Participants with chest x-ray CAD above threshold were also at a significant increased risk of tuberculosis, compared with those with CAD below threshold (Lunit ≥15 HR 11·39 [95% CI 4·48–28·93], CAD4TB ≥50 13·60 [5·34–34·64], and qXR >0·5 20·23 [7·90–51·82], p<0·0001) albeit with lower HRs than for participants with PET–CT scans consistent with tuberculosis (figure 3D; appendix p 23). In post-hoc sensitivity analyses, using different tuberculosis case definitions (culture-positive only, or including Xpert-positive untreated) had little effect on the HR for participants with PET–CT scans consistent with tuberculosis, whereas restricting to baseline QFT-positive status further increased the HR for these participants (appendix pp 8, 14–15, 23).

Given that not all individuals with PET–CT scans consistent with tuberculosis or inactive tuberculosis progressed to tuberculosis diagnosis, we next investigated whether there were PET–CT lesions or other demographic or clinical features differentiating those among them who did. To exclude any effect of previous tuberculosis on the lesion features investigated, we focused on the 30 of these participants with no previous tuberculosis: ten (33%) with a tuberculosis diagnosis and 20 (67%) with no diagnosis of tuberculosis over the study period (appendix p 16). Participants with a tuberculosis diagnosis were more likely to have baseline PET–CT scans consistent with tuberculosis than inactive tuberculosis (nine [90%] of ten *vs* six [30%] of 20, p=0·0052), cavities present (three [30%] *vs* none, p=0·030), more parenchymal lesions (median 6 [IQR 5–8] *vs* 2 [1–6], p=0·023), and infiltrates (1·5 [0–3] *vs* 0 [0–0], p=0·0016), and greater metabolic activity (maximal SUV_max_) of lung lesions (5·94 [5·21–8·96] *vs* 1·55 [1·22–1·92], p<0·0001). There was also a trend towards larger maximal size of parenchymal lesions (appendix pp 16, 24). Participants with a tuberculosis diagnosis also had lower baseline BMI, higher QFT nil, white cell counts, and neutrophil counts (p≤0·030). There was no difference in C-reactive protein, erythrocyte sedimentation rate, or QFT-stimulated values (appendix p 16). Sensitivity analysis restricted to participants with PET–CT scans consistent with tuberculosis including those with previous tuberculosis identified similar lesion FDG avidity and cell count differences and a higher erythrocyte sedimentation rate in those with a tuberculosis diagnosis than in those with no diagnosis (p≤0·030; appendix p 17).

**Figure 4:**
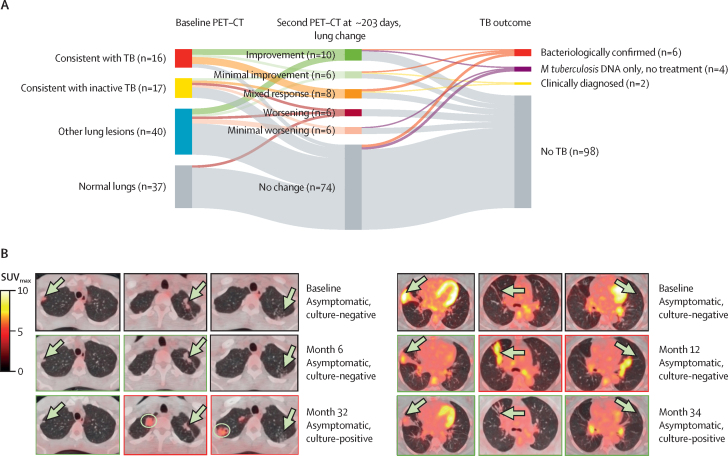
Lung parenchymal changes during follow-up compared with baseline PET–CT lung abnormality category and tuberculosis outcome (A) Plot linking individual participants' baseline PET–CT lung category to their summary change in lung lesions at the second PET–CT (after a median of 203 days [IQR 188–285]) and their end of study tuberculosis outcome. (B) Example axial sections of fused PET–CT change over time in two participants with baseline PET–CT scans consistent with tuberculosis who underwent three PET–CT scans: (left) one with improving (green outline) or unchanged lesions (black outline) at second PET–CT after 6 months who had lesions with a mixed response, showing worsening (red outline, with new lesions circled) and improving lesions at tuberculosis diagnosis at 32 months; (right) one who had all improving lesions at tuberculosis diagnosis at 34 months who has a mixed response of lesions at their second PET–CT at 12 months compared with baseline. Arrows point to lesions. The PET-CT colour scale indicates SUV_max_. Examples axial sections and all microbiological and chest x-ray results for all participants with *M tuberculosis* DNA detected or tuberculosis diagnosed during the study are shown in the appendix (pp 19–22). *M tuberculosis*=*Mycobacterium tuberculosis*. SUV_max_=maximum standard uptake value. TB=tuberculosis.

To understand the dynamic nature of lesions according to lung category following recent known tuberculosis contact, we compared the changes in parenchymal and lymph node lesions after a median of 203 days (IQR 188–285) in 110 participants who had repeat PET–CT without receiving tuberculosis treatment (appendix pp 6, 8, 25). They had a similar distribution of baseline lung categories as all 250 participants who had the first scan: 16 (15%) of 110 had PET–CT scans consistent with tuberculosis, 17 (16%) consistent with inactive tuberculosis, 40 (36%) showing other lung lesions, and 37 (37%) showing normal lungs (figure 4A). Parenchymal changes (improvement, worsening, or both [mixed response]) between scans were significantly more frequent in those with baseline PET–CT scans consistent with tuberculosis (12 [75%] of 16) than in those whose scans were consistent with inactive tuberculosis (four [24%] of 17), other lung lesions (seven [18%] of 40), or normal lungs (two [6%] of 37; p<0·0001; for LN changes, see appendix pp 8, 25). Eight (7%) of 110 participants who had a repeat PET–CT were subsequently treated for tuberculosis, after a median of another 669 days (IQR 222–842). Between baseline and repeat PET–CT (median 248 days [IQR 195–364]), two (25%) of these eight participants showed no change, three (38%) showed minimal improvement or improvement, and three (38%) showed a mixed response (figure 4A). The one participant with culture-positive tuberculosis at the time of repeat PET–CT was also the only one (1%) of 73 with baseline lesions to show complete resolution at repeat PET–CT (appendix p 20). Four of the eight participants with tuberculosis diagnosed after repeat PET–CT had a third scan at later tuberculosis diagnosis: two then showed improvement, one showed worsening, and one showed a mixed response compared with the second scan (figure 4B).

**Figure 5:**
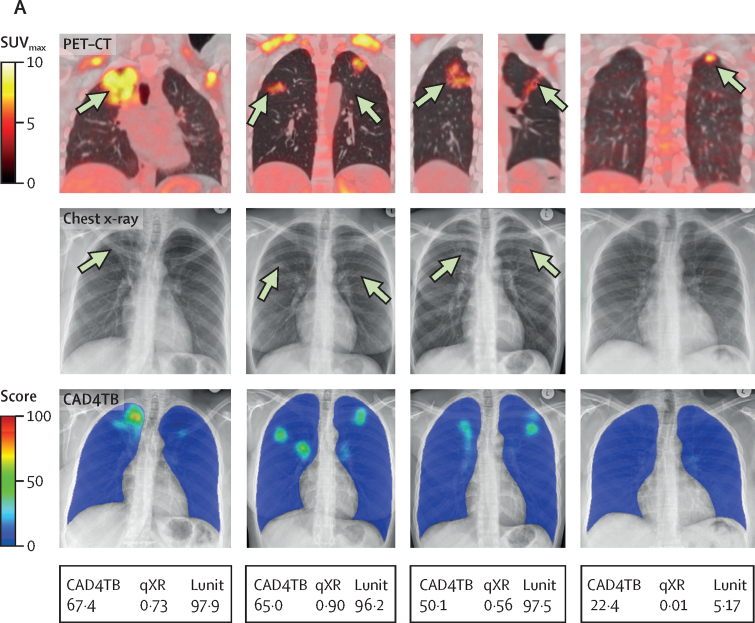

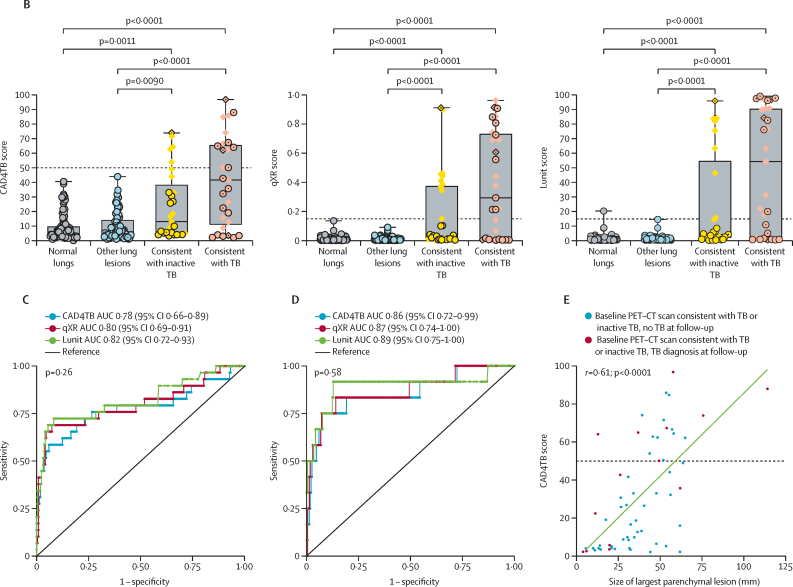
Evaluation of baseline CAD chest x-ray performance against baseline PET–CT (A) Radiographic images of four participants (one participant per column) with culture-confirmed tuberculosis at baseline where initial testing with Xpert MTB/RIF was either negative or the participant was unable to produce sputum spontaneously. The top row shows baseline fused PET–CT images of a single coronal section apart from the third participant from the left where two separate coronal slices are presented for the left and right lung separately as the main lesions could not be captured as a single section. All participants were classified as having PET–CT scans consistent with tuberculosis with lesions identified by the arrows. The PET-CT colour scale indicates SUV_max_. The middle row shows baseline chest x-rays. The three chest x-rays to the left were all reported as consistent with tuberculosis by a medical officer, with the final chest x-ray on the right reported as normal. The visible lesions are identified by the arrows. The bottom row shows CAD analysis of the baseline chest x-ray with CAD4TB used as an example. Blue signifies areas of normal lung, with green and yellow areas identifying areas of abnormality detected by the software, with CAD4TB score represented by the colour scale. The three chest x-rays on the left being above threshold and the one to the right were below threshold for all types of CAD software. (B) Box plots (line indicates the median, and whiskers indicate the IQR) showing distribution of CAD score by PET–CT status for the three CAD software types. Diamond symbols indicate participants with a history of tuberculosis, and internal dots indicate those with tuberculosis diagnosed and treated during the study. Dashed lines indicate CAD software threshold. p values were calculated using Kruskal–Wallis with Dunn's multiple comparisons test. (C) AUC analysis showing performance of CAD software against the reference standard of baseline PET–CT radiographically consistent with tuberculosis. p value for difference between the AUC tested by DeLong's method. (D) AUC analysis showing performance of CAD software against the reference standard of baseline PET–CT radiographically consistent with tuberculosis or inactive tuberculosis with subsequent bacteriological confirmation for tuberculosis at any timepoint over follow-up. p value for difference between the AUC tested by DeLong's method. (E) Scatter plot of CAD score against length of largest parenchymal lesion for 58 participants with PET–CT scans consistent with tuberculosis or inactive tuberculosis. The 14 participants subsequently bacteriologically confirmed at any timepoint over follow-up are shown in red, with the remainder in blue. The line of best fit is shown for all 58 participants. See the appendix for qXR and Lunit results (p 23) and Sankey diagrams linking participants by CAD thresholds and baseline PET–CT lung category to their final tuberculosis outcome (p 26). AUC=area under the receiver operator characteristic curve. CAD=computer-aided detection. SUV_max_=maximum standard uptake value. TB=tuberculosis.

Having found that most (14 [78%] of 18) asymptomatic individuals diagnosed with tuberculosis during the subsequent 5 years had baseline PET–CT scans consistent with tuberculosis or inactive tuberculosis, we next determined the performance of three types of commercially available CAD chest x-ray software implementable in high-burden settings to detect individuals with such lesions. Of the 249 of 250 baseline chest x-rays, valid CAD chest x-ray scores were obtained for 247 (99%) with CAD4TB and 248 (99%) with qXR and Lunit. Figure 5A compares examples of PET–CT, chest x-ray and CAD for the four participants who were baseline spontaneous sputum Xpert MTB/RIF-negative and bacteriologically confirmed by induced sputum culture. Across PET–CT lung categories, chest x-ray CAD scores were significantly higher in participants with PET–CT scans consistent with tuberculosis than in those with scans showing other lesions or normal lungs (p<0·0001) as was those with scans consistent with inactive tuberculosis (p≤0·009; table; figure 5B).

Of the 18 participants diagnosed and treated for tuberculosis, ten (56%), ten (56%), and eight (44%) had baseline chest x-ray-CAD scores below the tuberculosis-suggestive threshold for CAD4TB, qXR, and Lunit, respectively. Of these participants, six (60%) of ten, six (60%) of ten, and four (50%) of eight, respectively, had baseline PET–CT scans consistent with tuberculosis or inactive tuberculosis (appendix pp 8, 26). Using baseline PET–CT scans consistent with tuberculosis (n=29) as a radiological benchmark to evaluate CAD performance, AUCs were 0·78 (95% CI 0·66–0·89) for CAD4TB, 0·80 (0·69–0·91) for qXR, and 0·82 (0·72–0·93) for Lunit (figure 5C). Restricting the radiological benchmark to baseline PET–CT scans consistent with tuberculosis or inactive tuberculosis in participants with bacteriologically confirmed tuberculosis (n=12) improved chest x-ray-CAD diagnostic performance, with AUCs of 0·86 (95% CI 0·72–0·99) for CAD4TB, 0·87 (0·74–1·00) for qXR, and 0·89 (0·75–1·00) for Lunit (figure 5D). There was no significant difference between CAD software for either comparison (p≥0·26). In relation to this restricted benchmark, for chest x-ray CAD scores above the tuberculosis suggestive threshold, sensitivity was 58% (95% CI 28–85) and specificity was 95% (95% CI 92–98) for CAD4TB, 58% (28–85) and 97% (94–99) for qXR, and 75% (43–95) and 92% (87–95) for Lunit. There was a significant correlation between CAD score and maximal lesion size on PET–CT for CAD4TB, qXR, and Lunit (Pearson's *r*=0·56–0·61, p<0·0001; figure 5E; appendix p 23).

## Discussion

To our knowledge, this study is the largest to systematically use sensitive imaging in asymptomatic contacts of tuberculosis and the first with prolonged follow-up in the absence of treatment.^10^, ^18^ We found that 78% (14 of 18) of asymptomatic, HIV-uninfected adult tuberculosis contacts who were diagnosed with pulmonary tuberculosis over a 5-year period had PET–CT abnormalities reported as consistent with tuberculosis or inactive tuberculosis at baseline. Overall, of the 12% (29 of 250) of participants who had baseline PET–CT consistent with tuberculosis, 41% (12 of 29) were subsequently diagnosed with and treated for tuberculosis, with 60% (six of ten) of those who were culture positive having drug resistance or whole-genome sequencing concordant with the index case. We have shown that slow evolution of radiographically evident disease pathology can occur over many years without clinical manifestations and that short-term changes over 5–15 months do not relate to long-term outcome. All ten (100%) culture-positive participants with baseline PET–CT scans consistent with tuberculosis remained asymptomatic at the point tuberculosis was bacteriologically confirmed. By contrast, of the remaining six (of 16) participants with bacteriologically confirmed tuberculosis who did not have baseline PET–CT scans consistent with tuberculosis, all diagnosed after baseline, 83% (five of six) were symptomatic at diagnosis, including two who were QFT-negative at baseline but rapidly progressed to tuberculosis. This finding supports observations that different disease trajectories exist.^7^, ^8^, ^19^

The finding of slow, PET–CT-evident, asymptomatic disease evolution over many years mirrors the macaque experimental tuberculosis model in which a percolator phenotype has been described.^20^ We also extended our own observations and those of Kim and colleagues,^21^ who showed in a cohort of 20 people in the UK that PET–CT abnormalities in asymptomatic contacts were associated with DNA detection in blood, and Williams and colleagues,^22^ who showed in five people screened in South Africa with negative sputum Xpert MTB/RIF that PET–CT abnormalities were associated with detection of *M tuberculosis* DNA by facemask sampling.

Our findings have implications for tuberculosis prevention strategies because tuberculosis preventive therapy is usually recommended to asymptomatic individuals with evidence of immune sensitisation. In this instance, 82% (205 of 250) of QFT-positive asymptomatic contacts would have been treated with the possibility to prevent 89% of (16 of 18) cases of tuberculosis over 5 years. Conversely, focusing a treatment approach on just the 12% (29 of 250) of contacts with PET–CT evidence of tuberculosis at baseline would have prevented a maximum of 67% (12 of 18) of cases over the same period and avoided overtreating 97% (215) of the remaining 221. Notably, a meta-analysis comparing drug regimens to treat bacteriologically negative individuals with chest x-ray suggestive of tuberculosis found multidrug (three to four drugs) regimens were more effective than isoniazid-based regimes,^23^ suggesting that conventional tuberculosis preventive therapy would also be inadequate treatment for individuals with PET–CT scans consistent with tuberculosis. This finding is consistent with results of the CORTIS trial, in which 3 months of weekly isoniazid and rifapentine did not reduce tuberculosis progression in blood RNA tuberculosis biomarker-positive asymptomatic adults.^24^

Participants diagnosed with tuberculosis who had baseline PET–CT scans consistent with tuberculosis were all diagnosed within 3 years. Conversely, the two QFT-positive participants who were diagnosed with tuberculosis and were radiographically negative for tuberculosis or inactive tuberculosis at baseline were diagnosed after 32–53 months. This finding suggests that the current WHO target product profile^9^ for tests predicting tuberculosis progression that detect radiographically negative incipient tuberculosis that progresses within 2 years will not detect the majority of progressive tuberculosis in high-burden settings over this period and instead should focus on biomarkers for asymptomatic radiographically evident disease. Moreover, one of these two QFT-positive participants who did have PET–CT and chest x-ray repeated at 32 months when asymptomatically sputum culture positive then had PET–CT-evident tuberculosis. Therefore, consistent with historic community-wide screening approaches,^25^ this finding supports there being considerable time (eg, after 2–3 years) within which repeat radiographic (or potential biomarker) screening might be beneficial to detect asymptomatic tuberculosis development in those who are initially radiographically (or biomarker) negative. Moreover, the rapid progression trajectory of the two QFT-negative participants with a tuberculosis diagnosis also suggests that distinct biomarkers and follow-up timings will be required to detect all tuberculosis progressive states.

Although our findings show that PET–CT sensitively detects asymptomatic tuberculosis before sputum bacteriological confirmation, it is not a feasible routine screen. Chest x-ray is of lower resolution than PET–CT, and our study, to the best of our knowledge, is the first to evaluate chest x-ray CAD diagnostic performance against PET–CT, finding moderate performance for asymptomatic lesions consistent with tuberculosis (AUC 0·78–0·82). However, focusing the reference standard solely to participants with lesions that are ultimately sputum bacteriologically confirmed (most important to prevent transmission) improved the performance of CAD (AUC 0·86–0·89). This reflects our finding that participants who were eventually diagnosed with tuberculosis had significantly more lesions on PET–CT, with a tendency to larger maximum lesion size, which is more easily detectable by chest x-ray. This finding highlights the utility of chest x-ray as a key screening tool for asymptomatic tuberculosis but, as we have previously observed, the CAD cutoff thresholds used tend towards high specificity rather than sensitivity.^16^ Participants with PET–CT scans consistent with tuberculosis or inactive tuberculosis who were ultimately diagnosed with tuberculosis also had significantly higher blood neutrophil counts and lung maximum standardised uptake values, raising the possibility of future development of a host asymptomatic tuberculosis biomarker to complement or replace chest x-ray and, therefore, to facilitate more efficient scale-up of community-wide screening. Higher neutrophil counts also distinguish individuals with symptomatic tuberculosis from those with tuberculosis infection and cured tuberculosis.^26^

There are limitations to our study; it was conducted in a setting with a high tuberculosis burden in HIV-uninfected, adult contacts of patients with drug-resistant tuberculosis, and hence we can only speculate on how our findings relate to other populations. Ethnicity was not formally documented because residents of Khayelitsha are of predominant Black African Xhosa ancestry. Treatment decisions made following study investigations were deferred to local tuberculosis clinics, providing all study results, including routine PET–CT reports, when available. This might have biased decisions for the six (33%) of 18 direct referrals without culture confirmation. However, sensitivity analysis restricted to culture-confirmed cases yielded similar findings. Disease trajectories in the context of immunosuppression (eg, with HIV co-infection) differ, with potentially higher proportions of participants with asymptomatic tuberculosis progressing to symptomatic or bacteriologically confirmed tuberculosis. Some clinically important observations, including symptom status at microbiological confirmation, are based on small sample sizes, warranting confirmation in additional cohorts. In low-incidence settings, the likelihood of participants having previous tuberculosis or additional tuberculosis exposure during follow-up would be low. 14% of our cohort had previous tuberculosis, and residual changes of previously treated disease prove difficult to distinguish from changes associated with new disease, notwithstanding the median time since the end of previous tuberculosis treatment was about 9 years in participants with lung changes suggestive of tuberculosis. For these reasons, the performance of PET–CT might be better in a low-incidence setting.

Our results challenge current concepts of the progression of human tuberculosis and have implications for diagnostic, prognostic, and intervention strategies. The majority of the 5-year disease risk in people with a clinical diagnosis of tuberculosis infection is borne by those with existing radiographically evident disease. CAD chest x-ray performed well in detecting participants with the most clinically significant lesions on PET–CT. Although scaling up symptom-agnostic community-wide active case finding with chest x-ray has additional challenges, it also offers real opportunity for tuberculosis elimination.^25^ Further development of diagnostics and therapeutic approaches are needed to better target this population and reduce the numbers needed to treat to prevent a case of clinical disease. Such tuberculosis care and prevention strategies could greatly limit onwards transmission and potential post-tuberculosis sequelae.

### Contributors

### Data sharing

Requests for clinical metadata will be reviewed by AKC, HE, and RJW to determine whether the request is subject to confidentiality and data protection obligations, upon reasonable request. Data that can be shared will be released via a data sharing agreement. *M tuberculosis* whole-genome sequencing data is deposited in the NCBI Sequence Read Archive, which is accessible via the BioProject accession number PRJNA1438398. TBtypeR is published,^27^ and the is code available online.

## Declaration of interests

AKC reports grant funding from the South African Medical Research Council, US National Institutes of Health (NIH), the Gates Foundation, Wellcome, UK Research and Innovation (UKRI) Medical Research Council (MRC), Australian National Health and Medical Research Council (NHMRC), and the Walter and Eliza Hall Institute of Medical Research (WEHI); provision of QFT reagents from QIAGEN; and travel support from WHO. CRH reports participation on data safety monitoring boards for the SUDOCU, ACT5, and DECISION trials, serving as Chair of the trial steering committee for UNITE4TB, and serving on the Board of Directors of the International Union Against Tuberculosis and Lung Disease (2019–25). CRH, DA, JJE, JLF, and PS report grant funding from the US NIH. DA receives royalty payments, equipment, and supplies and research grants from Cepheid, and equipment and supplies from Molbio. GW reports grant funding from the US NIH, the Gates Foundation, and the European and Developing Countries Clinical Trials Partnership. HE reports grant funding from UKRI and UK MRC and Wellcome, consulting fees from Evidera (PPD), and participation on data safety monitoring boards for the US National Institute of Allergy and Infectious Diseases (NIAID) and StatinTB trials. LEV reports travel support from the Gates Foundation. RJW reports grant funding from the South African Medical Research Council, US NIH, Wellcome, UK MRC, and Cancer Research UK. RMW reports salary from the South African Medical Research Council. All other authors declare no competing interests.
