# Supplementary material for: PET–CT benchmarked detection and 5-year progression of asymptomatic tuberculosis: a longitudinal, prospective cohort study
Source: Lancet Respir Med. 2026 Jun;14(6):505–20. doi: 10.1016/S2213-2600(26)00056-1 (PMC13229962; doi:10.1016/S2213-2600(26)00056-1)
Supplement: Supplementary appendix [file mmc1.pdf]

THE LANCET
Respiratory Medicine

Supplementary appendix

This appendix formed part of the original submission and has been peer reviewed. We post it as supplied by the authors.

Supplement to: Esmail H, Thienemann F, Sossen B, et al. PET–CT benchmarked detection and 5-year progression of asymptomatic tuberculosis: a longitudinal, prospective cohort study. *Lancet Respir Med* 2026; published online March 23. https://doi.org/10.1016/S2213-2600(26)00056-1.

PET/CT benchmarked detection and 5-year progression of asymptomatic tuberculosis: a longitudinal, prospective cohort study

SUPPLEMENTARY APPENDIX

SUPPLEMENTARY METHODS 3

DETAILED DESCRIPTION OF RECRUITMENT, CONSENT AND PROCEDURES 3
REPEAT FDG-PET/CT 3
FOLLOW UP FOR TB 3
QUANTIFERON ASSAYS 4
CHEST RADIOGRAPH READING 4
ADDITIONAL CAD METHODOLOGY 4
FDG-PET/CT 5
PERFORMING FDG-PET/CT SCANS 5
READING FDG-PET/CT SCANS 5
RADIOGRAPHIC CATEGORIZATION OF PARTICIPANTS 6
CATEGORIZATION OF CHANGE BETWEEN BASELINE PET/CT SCAN AND FOLLOW-UP PET/CT SCAN 6
BRONCHOSCOPY 6
SPUTUM PROCESSING FOR *MYCOBACTERIUM TUBERCULOSIS* 7
MYCOBACTERIUM TUBERCULOSIS CULTURE FOR DNA EXTRACTION AND EXTENDED DRUG SENSITIVITY TESTING 7
MYCOBACTERIUM TUBERCULOSIS WHOLE GENOME SEQUENCING (WGS) 7
MTB WGS DATA PROCESSING AND ANALYSIS 7

SUPPLEMENTARY RESULTS AND DISCUSSION 8

TB RISK SENSITIVITY ANALYSES 8
FDG-AVID LYMPHADENOPATHY 8
COMPARISON OF CAD-CXR AGAINST DIAGNOSED AND TREATED TB 8
BRONCHOALVEOLAR LAVAGE CULTURE 8
DRUG SUSCEPTIBILITY RESULTS AND INDEX CASE LINKAGE 9

SUPPLEMENTARY TABLES 10

Table S1. Demographics of adult TB contacts who underwent and those excluded from, PET/CT. 10
Table S2. Distribution of lesion types and spatial location (bronchopulmonary segment) by baseline PET/CT radiographic category. 11
Table S3. Baseline characteristics, clinical and radiographic findings, and TB outcomes of household TB contacts who underwent PET/CT with no previous TB diagnosis. 12
Table S4. Microbiological and clinical characteristics of participants with culture positive TB or only Xpert positive samples, compared to their index case drug sensitivity profile. 13
Table S5. Univariate and multivariate analyses of the risk to develop TB over the study periods due to baseline PET/CT lung parenchymal category and main covariates. 14
Table S6. Characteristics of participants with PET/CT consistent with TB or inactive TB with no previous TB history in relation to 5-year TB diagnosis and treatment 16
Table S7. Characteristics of participants with PET/CT consistent with TB (including those with previous TB history) in relation to 5-year TB diagnosis and treatment. 17

SUPPLEMENTARY FIGURES 18

Figure S1. Demographic, blood, and radiographic findings by baseline PET/CT lung classification 18

Figure S2. Radiographic and microbiological findings at baseline and TB diagnosis or follow-up. 19-22

Figure S3. Baseline PET/CT lung category and chest radiograph computer-aided detection (CXR-CAD) scores in relation to TB outcome over study period and CXR-CAD scores in relation to size of largest baseline PET/CT parenchymal lesion. 23

Figure S4. Distribution, size, type and metabolic activity of lesions on baseline scan in those with PET/CT consistent with TB and no previous history of TB by microbiological status. 24

Figure S5. Lung parenchyma and lymph node changes between baseline and second PET/CT, according to baseline PET/CT lung category 25

Figure S6. Sanky diagrams linking participant chest radiograph computer-aided detection (CXR-CAD) thresholds with baseline PET/CT lung category to their final TB outcome 26

REFERENCES 27

Supplementary Methods

Detailed description of recruitment, consent and procedures

All participants were residents of Khayelitsha, a peri-urban township of Cape Town, South Africa, where TB incidence at the time of study initiation was > 900/100,000, and >95% of residents are of Black African Xhosa ancestry. Index cases aged ≥15 years with at least rifampicin resistant (RR) pulmonary TB confirmed by Xpert MTB/RIF or culture consented to a household visit and to contact individuals who met the definition of a HHC. At that visit a detailed description of the household's setting and composition were taken. Listed individuals who met the definition of a HHC at least 18 years of age interested in participation were given an appointment at the study clinic for screening.

Following initial consent at the screening visit, all HHC underwent medical history and examination, sex was self-reported, screening for HIV with point of care testing followed by HIV ELISA if negative, screening for TB with symptom screen aligned with the South African National Guidelines (unexplained cough for 2 weeks, fever, night sweats, weight loss), physical examination for signs of pulmonary and extrapulmonary TB, digital posterior anterior (PA) chest radiography (CXR) and three sputum samples (the first spontaneously produced if able with the remainder induced using nebulised 3% saline to a total of three) all three being sent for smear, Xpert MTB/RIF and culture irrespective of symptoms or CXR findings. In addition, a random capillary plasma glucose was taken to screen for diabetes mellitus and in women of child bearing potential a urine sample was taken for pregnancy testing.

Of those screened a subgroup of 250 further consented to undergo FDG-PET/CT subject to the following pre-specified inclusion/exclusion criteria

- Inclusion criteria - age ≥ 18 years, willing to undergo HIV counselling and testing (HCT)
- Exclusion criteria - HIV infection, on TB treatment at the time of screening, symptoms or signs of active TB or acute illness (one or more of the following symptoms: unexplained cough > 2 weeks, unexplained fever >1 week, night sweats, unintentional weight loss, pleuritic chest pains, haemoptysis, fatigue or shortness of breath on exertion; new onset of symptoms deemed by study physician to be possibly related to active tuberculosis or acute illness. If symptoms resolve and all other clinical indicators are negative for TB, the contact could be rescreened for eligibility), age >65 years, smoker >30 pack years, diagnosis of malignancy, diagnosis of chronic lung infection other than TB (e.g., non-tuberculosis mycobacteria [NTM], Fungal), diagnosis of chronic inflammatory condition associated with pulmonary pathology (e.g., Sarcoid, Rheumatoid arthritis, Granulomatosis with polyangiitis, bronchiectasis), inhaled or systemic steroid use within previous 2 weeks, breast feeding, pregnant, or planning pregnancy over next 3 months, unable to be followed up for 6 months, or uncontrolled diabetes mellitus.

Five eligible individuals were excluded in a protocol deviation, based on investigator's initial interpretation of index case microbiological results indicating contact non-eligibility.

The 250 participants undergoing FDG-PET/CT scanning also consented to additional blood sampling for full blood count (FBC), C-Reactive Protein (CRP), Erythrocyte Sedimentation Rate (ESR), QuantiFERON-TB Gold in tube (QFT-Gold), and QuantiFERON-TB Gold Plus (QFT-Plus). Bloods for additional immunological investigations, urine and sputum were processed and stored, with TB biomarker and immunological responses to *Mtb* infection related secondary outcomes to be reported separately in the future.

Repeat FDG-PET/CT

112 of the 250 participants provided further consent to repeat FDG-PET/CT imaging performed at 5-15 months, including two diagnosed and treated at baseline. 110 therefore underwent a second PET/CT without treatment. All participants in order of recruitment were invited to undergo a second scan, until approximately 100 were complete. We then enriched the sample with those who were either QFT negative with normal baseline scans and invited all with lesions consistent with TB to ensure the cohort undergoing repeat scans was well distributed across lesion phenotypes. All those undergoing repeat FDG-PET/CT imaging had a sputum sample (induced if needed) sent for smear, Xpert MTB/RIF and culture. In addition, blood was taken and processed similarly to the baseline visit. Choosing to decline the repeat PET/CT was not an exclusion criterion for clinical follow-up.

Follow up for TB

All participants were contacts of drug-resistant TB and, in line with South African and WHO recommendations which reflect the limited evidence base, preventive therapy was not offered with participants instead monitored closely for the development of active TB with referral to specialist services upon concern. Follow up of the entire

screened cohort was both active and passive. The 250 participants were contacted by phone or SMS approximately every 3 months and advised to attend the research clinic for investigation if they had symptoms or signs of TB. They were seen in person at the research clinic at approximately 12 months (range 9-16m) and assessed clinically for signs and symptoms of TB with sputum investigations conducted if symptomatic. All participants screened at baseline, including those who did not undergo FDG-PET/CT were seen at the clinic between 23 to 38 months and again actively screened for TB with three sputum samples (induced if needed) sent for smear, Xpert MTB/RIF and culture (irrespective of symptom status) and bloods taken for FBC and QFT-Gold and -Plus. In addition, all participants consented to inspection of their health service encounters via the Provincial Health Data Centre (PHDC) and clinic medical records to ensure that all episodes of TB that were diagnosed and/or treated within the Western Cape Province were captured.[1] The search of the PHDC record took place on 21st May 2021 to capture all TB episodes from the start of the study to this date and to confirm previous episodes prior to study enrolment.

Any of the 250 participants undergoing baseline FDG-PET/CT scan who developed TB during follow up were invited for further assessment which included history, examination, CXR, bloods and repeat FDG-PET/CT provided this did not result in more than 2 scans occurring within a 12 month period in order to limit exposure to ionising radiation.

Participants with positive microbiological research investigations and those with persistent TB signs and symptoms but negative on microbiological research investigations were referred to the local TB clinic with relevant results and the final decision to treat and drug regimen was determined by the statutory TB service following their own investigations. Where possible medical teams in the statutory health service were provided with relevant results of clinical investigations from the study to assist them with decision making in the participants interests which could include microbiology and laboratory results and routine PET/CT clinical reports, if available, but not the final PET/CT status.

QuantiFERON assays

The QFT-Gold and QFT-Plus assays (QIAGEN, Valencia, CA) were conducted and scored in accordance with manufacturer's instructions, with the addition of performing an 8-point 1/2 dilution series, to improve IFN-ɣ ELISA standard curve accuracy. For samples where the antigen stimulated IFN-ɣ value was >10 IU/ml and could not be accurately extrapolated from the standard curve, plasma was diluted up to 1/100 until a quantifiable result was obtained.

Chest radiograph reading

CXR were performed using a digital X-Ray machines (Delft Oldeca DR or Phillips Essenta DR) and captured posterior-anteriorly in full inspiration with the participant standing. The Digital CXR images were viewed on 2 megapixel screens using the OsiriXMD version 11.0.4 (Pixemo, Bernex, Switzerland) software package and reported by a medically qualified investigator blinded to clinical and microbiological status. CXR were fully assessed for evidence of TB and then classified as consistent with active TB, inactive TB, abnormal but not consistent with TB, or normal.

Additional CAD methodology

In exploratory post-hoc analysis, three CAD software were evaluated: CAD4TB version 7.0 (CAD4TBv7, Delft Imaging, 's-Hertogenbosch Netherlands), qXR version 3.0.0 (qXRv3, qure.ai, Mumbai, India) and Lunit INSIGHT CXR version 3.1.4.111 (Lunitv3, Lunit, Seoul, South Korea). CAD software was installed by the companies on local servers (one for each software) under the control of FIND, one of the investigator organisations in this study. After successful installation, access to the servers was removed from the CAD vendors to allow independent evaluation. Anonymised digital images were uploaded via a secure server to FIND and then processed by the CAD software. No images were shared with the software manufacturers. The CAD output consists of a score along a continuous scale (CAD4TB and Lunit INSIGHT CXR 0-100, qXR 0-1), where high scores indicate a higher probability of active TB. For analysis, for qXRv3 and Lunitv3 the manufacturer recommended threshold scores of 0.5 and 0.15 respectively were used and for CAD4TBv7 where the manufacturer does not specify a threshold a commonly used threshold of 50 was used.

FDG-PET/CT

Performing FDG-PET/CT scans

FDG-PET/CT scans were performed at three different sites, all within approximately 15 miles from the study site in Khayelitsha; the Cape PET-CT centre in Panorama Mediclinic using a Siemens Biograph PET/CT machine, the Western Cape Academic PET-CT centre at Tygerberg provincial hospital using a Phillips Gemini PET/CT machine or the Cape Universities Body Imaging Centre (CUBIC) using a Siemens Biograph PET/CT machine. Imaging protocols were similar (PET Parameters: 120 kV, 200 mA, 0.75 seconds rotation time, and a pitch of 0.438 with a collimation of 16x0.75 mm. CT Parameters: 110 KV/200 Ma, I31s, Strength 3). All repeat imaging was performed on the same machine as the initial scan.

Participants undergoing FDG-PET/CT fasted for six hours prior to the scan and were escorted to the PET/CT centre by a research worker. Point Of Care (POC) blood sugar was performed and if ≥11.1mmol/L the scan was rescheduled. 2.8MBq/kg of FDG was administered via a cannula. Sixty minutes after FDG administration the PET/CT scan was performed. CT was limited to the thorax (neck to liver) to reduce radiation exposure. Total effective radiation dose per scan was approximately 10mSv (varying with body weight and height). The second PET/CT was performed on the same scanner as the first PET/CT using a similar dose of FDG and injection to scan time as the initial scan.

Reading FDG-PET/CT scans

PET/CT scans were read by two independent readers who provided independent structured reports focusing on the lung parenchyma plus mediastinal and hilar lymph nodes, with differences resolved by a third reader. All were blinded to clinical history and microbiological results. Detailed reporting instructions were provided to all readers. One report was provided by a nuclear medicine physician/radiologist and the second by infectious diseases physician experienced in the reporting and analysis of research PET/CT scans for tuberculosis. Differences between the two structured reports were resolved by a 3rd reader also blind to clinical history and microbiological results. The nuclear medicine physician/radiologist in addition provided a full clinical report to comment on additional findings.

Parenchymal lesions were categorised as infiltrates, fibrotic scars, apical scarring, active nodules or discrete nodules according to prespecified definitions below:

- Infiltrate: Irregular, air space opacification which may include presence of clusters of micronodules, tree-in-bud appearance or denser consolidation.
- Fibrotic scar: Linear fibrotic or fibro-cystic abnormalities slightly distorting the surrounding lung tissue with no radiographic signs of activity which could be calcified with only minimal FDG uptake.
- Apical scarring - minimal subpleural scars typically less than 10 mm long at the apex of upper lobe was distinguished from fibrotic scars.
- Nodules: Near-spherical opacities within the lung parenchyma which may have well- or poorly-defined edges being less than 3 cm in diameter, and typically 3-10 mm in size,
- Other lesions: Any other abnormality found in the lung parenchyma or pleura was captured as “other”. In particular bronchiectasis, ground glass opacification and pleural thickening was recorded.

The size and location of lesions (bronchopulmonary segment) were described. The lesions were also evaluated for radiographic signs of disease activity (e.g. cavitation, tree-in-bud appearance, poorly defined margin). The density of the lesion was assessed by maximum Hounsfield Units (HU). FDG uptake within the parenchyma lesions was quantified by maximal standardised uptake value (SUV_{max}) and Visual Score (VS) (VS = 0 – No visible uptake of FDG, VS = 1 – FDG uptake within lesion greater that background lung parenchyma but less than mediastinal blood pool, VS = 2 – FDG uptake within lesion greater than mediastinal blood pool but less that liver, VS = 3 – FDG uptake within lesion greater than liver). Parenchymal lesions were considered to have abnormal FDG uptake if VS was ≥ 1. SUV_{max} and VS of mediastinal and hilar lymph nodes was assessed and FDG uptake was considered abnormal if VS ≥ 2. Mediastinal and hilar LN were also assessed for size (considered abnormal if the short axis width was greater than 1 cm) and for evidence of mineralization. Abnormal lymph nodes were placed into one of the following lymph node basins following convention of the International Association for Study of Lung Cancer (IASLC); right or left superior mediastinal (IASLC 2 – 4), aortic (IASLC 5, 6), subcarinal (IASLC 7), inferior mediastinal subcarinal (IASLC 8, 9) and right or left hilar (IASLC 10 –14).

Radiographic categorization of participants

Following the consensus reading process outlined above, the baseline FDG-PET/CT scans were classified into the prespecified, mutually exclusive lung categories below on the basis of the radiographic findings alone. These definitions were based on findings from our prior work which in turn reflect findings from previous imaging and autopsy studies on findings in early TB disease.[2-4] In our previously published study in people living with HIV-1[2] we grouped together those with infiltrates, active nodules and fibrotic scars into a single asymptomatic TB category (subclinical TB). In this study which had a larger sample size we separated those with infiltrates and active nodules from those with only fibrotic scars into 2 subgroups (Radiographically consistent with TB or Inactive TB, respectively) to better understand the trajectory and prognosis of these pathologies.

- Radiographically consistent with TB disease (PET/CT-TB) - Presence of infiltrate(s) in bronchopulmonary segment R1, R2, R3, R6, L1/2, L3 or L6 **OR** Presence of nodule(s) with VS ≥1 in bronchopulmonary segment R1, R2, R3, R6, L1/2, L3 or L6
- Radiographically consistent with inactive TB (PET/CT-inactive TB)- Presence of fibrotic scar(s) (**NOT** isolated apical scar) in bronchopulmonary segment R1, R2, R3, R6, L1/2, L3 or L6
- Other lung abnormalities of uncertain significance (PET/CT-other lung lesions) - Infiltrates, scars or nodules in other bronchopulmonary segments **OR** other parenchymal lesions (e.g. bronchiectasis)
- Normal lung parenchyma (PET/CT-normal lung) – No radiographically visible lesions within the lung parenchyma. This group may have mediastinal and/or hilar lymph node abnormalities.

Metabolically active (FDG-avid) Lymph Nodes were defined as lymph nodes of any size with VS ≥ 2 in the mediastinal, hilar or aortic location. Hot lymph nodes could be present alone or in combination with any of the above parenchymal categories

Categorization of change between baseline PET/CT scan and follow-up PET/CT scan

The change between the baseline and follow-up scan was determined for parenchymal lesions and lymph node lesions separately and classified as follows:

- **No change** – No change in visual score of any lesions between baseline and follow-up scan
- **Minimal change** – Single lesion change in visual score ± 1 between baseline and follow-up scan
- **Improvement** – Single lesion reduction in visual score of >1 **OR**
  considerable reduction in size or SUVmax (as determined by reader) if visual score = 3 **OR**
  >1 lesion reducing by visual score = 1 **OR**
  complete resolution of lesion between scans
- **Worsening** – Single lesion increasing in visual score of >1 **OR**
  considerable increase in size or SUVmax (as determined by reader) if visual score = 3 **OR**
  >1 lesion increasing by visual score = 1 **OR**
  new lesion(s) developing between scans
- **Mixed** – Some lesions worsening while others improving

Bronchoscopy

To confirm whether *Mtb* could be isolated more frequently from PET/CT lesions in the lung consistent with TB/inactive TB compared to lungs without such lesions, HHC with such abnormalities on baseline or repeat PET/CT or those who fit criteria to undergo a control bronchoscopy were asked to return to the study clinic up to 8 weeks after the repeat PET/CT once the structured report of the repeat PET/CT was available. Those with PET/CT abnormalities consistent with active TB at either baseline or repeat PET/CT were referred for Bronchoscopy unless contra-indicated, following additional consent procedure. It was estimated 30 participants of the estimated 50 who would have active PET/CT abnormalities would be eligible for bronchoscopy. Thirty of an age and sex matched group of 50 participants without active PET/CT abnormalities would be approached to provide additional consent to have research bronchoscopy performed following repeat PET/CT. In total, 28 participants consented and underwent bilateral PET-CT lesion guided bronchoscopy, performed at Tygerberg Hospital. 150ml of saline was first instilled into the contralateral lobe to the region of interest, followed by 150ml instilled into the lobe with lesions of interest. Approximately 50-70ml bronchoalveolar lavage fluid (BALF) was obtained from each lobe, and 10 ml from each lobe was sent for MGIT culture in the TB Genomics Laboratory, Tygerberg Campus, Stellenbosch University. Cultures were incubated for 42 days before being classified as negative.

Sputum processing for *Mycobacterium tuberculosis*

All sputum samples were processed in the accredited laboratories of the South African National Health Laboratory Services (NHLS) where auramine sputum smear, Xpert MTB/RIF (Cepheid, Sunnydale, CA) and mycobacteria growth indicator tube (MGIT) liquid TB culture (BD Diagnostic Systems) were performed. Cultures were incubated for 42 days before being classified as negative.

Mycobacterium tuberculosis culture for DNA extraction and extended drug sensitivity testing

Following collection from the NHLS, culture positive MGIT tubes were transported to the Biosafety Level 3 (BSL3) Laboratory of the Institute of Infectious Disease and Molecular Medicine, University of Cape Town. MGIT cultures were pelleted and *Mtb* was resuspended in 2ml 7H9/25% glycerol and two 1 ml aliquots stored at -80°C. Stocks were prepared for DNA extraction and identification of rifampicin (RIF) resistant colonies as previously described[5]. Briefly, glycerol stocks were inoculated in 5 ml 7H9/ADC/0.05 % Tween 80 and incubated at 37°C, once they reached confluent growth after approximately 7-10 days, 500 µl of broth culture was plated on two 120 mm 7H10/OADC plates and one 7H10/OADC plate containing 1 µg/ml RIF (7H10/RIF) for selection of resistant single colonies. Plates were incubated at 37°C for up to 8 weeks and growth monitored. *Mtb* which grew on plates without RIF were scraped into one 50ml falcon containing 3 ml sterile H_2O, once confluent. *Mtb* was heat-killed at 80°C in a water bath for 1 hour, followed by phenol/chloroform DNA extraction, as previously described[6]. Cultures which grew on 7H10/RIF plates were recorded as RIF resistant and individual *Mtb* colonies which grew on 7H10/RIF were picked and inoculated into 5 ml 7H9/ADC/0.05 % Tween 80 containing 1 µg/ml RIF and incubated at 37°C. Once they reached confluence, 500 µl of broth culture was plated on two 7H10/OADC plates and plates incubated until confluent growth. To make a stock of the 7H9 RIF culture, 500 µl of the 7H9 broth culture was added to 500 µl of 50% glycerol and stored at -80°C. Once plates were confluent, they were scraped and heat-killed and DNA extracted as above.

For extended drug sensitivity testing, glycerol stocks from the original MGIT bulk cultures and those made from the RIF resistant colonies grown in 7H9 were sent to the TB Genomics Laboratory, Tygerberg Campus, Stellenbosch University. Phenotypic drug sensitivity was performed using the Sensititre MYCOTB MIC test (Thermo Scientific) for 12 first- and second-line anti-TB drugs: amikacin, cyclosporin, ethambutol, ethionamide, isoniazid, kanamycin, moxifloxacin, ofloxacin, para-aminosalicyclic acid, rifabutin, rifampicin, and streptomycin. Pyrazinamide susceptibility testing was performed using the BACTEC MGIT 960 method (BD Diagnostics).

Mycobacterium tuberculosis whole genome sequencing (WGS)

Prior to library preparation, DNA samples were cleaned by adding 50 µl genomic DNA solution with 40 µl (0.8x) magnetic beads (NucleoMag®). Samples were incubated for 5 minutes at room temperature (RT) to bind the DNA and then washed with 200 µl 80% ethanol twice while on a magnetic stand. Cleaned genomic DNA was eluted in 15 µl of nuclease-free water (Ambion). One hundred ng of cleaned DNA was fragmented using Fragmentase (NEB) following manufacturer's instructions with some modifications. Briefly, to fragment the genomic DNA, 20 ul solution containing DNA, *Fragmentase*, 10x buffer and $MgCl_2$ were incubated at 37 °C for 30 mins on a PCR machine. Enzymes were deactivated at 65 °C for 30 minutes. Unbound excess reagents were removed by diluting the sample to 40 µl with nuclease-free water followed by a bead clean-up using 1.5x volume of magnetic beads (NucleoMag®) and two washes of 80% ethanol. Finally, bound fragmented DNA was eluted in 65 µl of nuclease-free water. Sixty µl of fragmented genomic DNA (100 ng) was indexed for Illumina sequencing using the TruSeq DNA sample Prep Kit (Illumina) per manufacturer's instruction. Library concentration was quantified by Qubit™ dsDNA Assay kit and Picogreen Assay (Thermo Fisher Scientific) and library size was determined using TapeStation (Agilent). Equimolar amounts of libraries were pooled and diluted to 750pM for 150-bp paired-end sequencing on a NextSeq2000 instrument using the P2 300- cycle kit v2 chemistry (Illumina) as per manufacturer's instructions. To produce the sequences the base calling and quality scoring was performed by the Real Time Analysis (v2.4.6) software. The FASTQ file generation and de-multiplexing for the samples was performed by the bcl2fastq conversion software (v2.15.0.4).

Mtb WGS data processing and analysis

Alignment and variant calling were performed according to the GATK4 Germline short variant discovery best-practices workflow with joint-calling[7] against the *Mycobacterium tuberculosis* H37Rv reference genome (NC_000962), with the omission of "base quality score recalibration" and "variant quality score recalibration" steps. SNP variant calls were initially QC filtered based on the following VCF annotations: MQ > 30, FS < 60, SOR < 3, MQRankSum > -12.5, ReadPosRankSum > -8. To calculate pairwise SNP distances, the SNP variant callset was further filtered by first removing all samples with > 5% missing variant calls or median depth < 10X,

then by removing variants with > 5% missing calls or median depth less than 3 standard deviations below the mean median variant depth. *Mtb* sublineages, mixed infections and drug resistance were then detected by an in-development software package TBtypeR[8]. Briefly, TBtypeR examines variant allele frequencies at a panel of lineage specific variant sites compiled from Napier et al.[9], Thawornwattana et al.[10], Coscolla et al.[11] and Shuaib et al.[12] to assign *Mtb* sublineage and mixture frequencies. Following this, variant allele frequencies at known drug resistance sites derived from The 2021 WHO catalogue of *Mycobacterium tuberculosis* complex mutations associated with drug resistance[13] was examined to assign drug resistance prediction.

Supplementary Results and Discussion

TB risk sensitivity analyses

The primary analysis of factors associated with risk of TB diagnosis used the TB case definition of being diagnosed and treated for TB (n=18). Sensitivity analyses (see appendix pp 14-15, 23) using a more restrictive (culture only, n=14) or expanded (all case including those treated and those untreated Xpert-positive culture negative, n=22) TB case definition had little impact on PET/CT-TB HR, 22.98 (95% CI 5.02-105.19) and 20.59 (5.85-72.43), respectively, compared to the primary analysis HR of 28.54 (6.37-127.81). The expanded definition found PET/CT-inactive TB also with significant HR 4.88 (1.09-21.80) (p=0.038). The HR for all case definitions were also increased with adjustment for previous TB. Post-hoc sensitivity analysis focusing on those treated for TB (primary definition) who were known QFT+ at baseline (n=16) (i.e. excluding those without evidence of *Mtb* infection at baseline), increased PET/CT-TB HR to 43.14 (5.60-332.56) and with adjustment for previous TB to the highest aHR of 71.94 (9.06-571.07), p<0.0001.

Although the decision to treat was not taken by investigators, we did provide results of some investigations to the physicians at the statutory health clinics, where this was feasible, in the participants interests. We cannot exclude that this might have influenced treatment decisions however our sensitivity analyses showing the use of different diagnostic definitions had limited effect on PET/CT-TB HR shows that the impact of this was likely to be minimal. The statutory health clinics repeated microbiology testing to inform their treatment decision.

FDG-avid lymphadenopathy

53/248 participants (21%) had FDG-avid mediastinal and hilar lymph nodes (LN) (Table 1). The distribution of FDG-avid LN between LN stations was Hilar LN 55%, Superior mediastinal LN 23%, Aortic LN 8%, Subcarinal LN 10%, Inferior mediastinal 2%. Whilst there was no significant difference in LN station distribution by baseline lung category (p=0.14), the presence of FDG-avid LN was significantly different, found in 16/29 (55%) PET/CT-TB, 12/30 (40%) inactive TB, 15/83 (18%) other lesions, and 10/108 (9%) normal lungs (p<0.0001, Table 1) and more likely in those with PET/CT-TB/inactive TB than without (48% [28/58] vs. 13% [25/190], p<0.0001). LN SUV_{max} was also significantly higher in those with PET/CT-TB compared to other lesions or normal lung (p=0.02, Table 1).
Between baseline and repeat PET/CT there was no significant difference in noticeable changes in lymph nodes related to baseline lung category (p=0.136) (appendix pp 25).

Comparison of CAD-CXR against diagnosed and treated TB

Using manufacturer recommended or commonly used CAD thresholds for CXR findings suggestive of TB scored 18/247 (7%) with CAD4TBv7≥50, 14/248 (6%) with qXRv3≥0.5, and 29/248 (12%) with Lunitv3≥15 (Figure 5B). CXR-CAD scores were above threshold for 5/6 (83%) participants (all software) with baseline bacteriologically-confirmed TB, median (IQR) scores CAD4TBv7 66.2 (50.1-88.0), qXRv3 0.77 (0.56-0.91), Lunitv3 97.2 (96.2-97.9). However, CXR-CAD scores were only above threshold in 3/12 (25%) (CAD4TBv7 and qXRv3) or 5/12 (42%) (Lunitv3) with TB diagnosed during follow-up (appendix p 26).

Bronchoalveolar lavage culture

In total, 28 participants underwent bilateral bronchoscopy, 12 with PET/CT consistent with TB or inactive TB, 6 with other lung lesions and 10 individuals with normal lungs; first sampling the uninfected lobe(s) and then the contralateral lobe with evident lesions, when present. All samples were culture negative. Of the 12 with lesions consistent with TB/inactive TB, one was subsequently treated for symptomatic TB after a further 14 months, and one was sputum Xpert-positive culture-negative after a further 11 months, whilst remaining asymptomatic. Both had worsening TB lesions on second PET/CT prior to bronchoscopy. One further participant with inactive TB lesions at baseline, when then Xpert-positive culture-negative, had BAL performed after 13 months and remained

culture negative in absence of treatment (appendix pp 21-22). Previous studies have similarly shown that BAL does not provide increased sensitivity compared to sputum induction.[14]

Drug susceptibility results and index case linkage

Importantly, the purpose of our study was not to explore the natural history following transmission but to use those with a recent known contact with DR-TB in a high burden setting as a way to identify many high-risk individuals to determine the natural history of baseline-evident asymptomatic disease irrespective of the source, by enrolling individuals for whom TB prophylaxis couldn't be provided at the time of the study. Contact-Index linkage information is only provided for completeness. Drug-resistance profiles for all case are summarised in appendix pp 13 and 19-22.

In a high burden setting we cannot assume that exposure to the index case is the first ever *Mtb* infection event of the HHC, it just represents a recent known exposure. We in fact hypothesised that it is highly likely that some HHC would be infected at baseline from a distant transmission event. This is supported by the fact that only 4/6 (67%) baseline cases were diagnosed with DR-TB and 2/6 (33%) had drug-sensitive TB (DS-TB). It could be possible that the four most distantly diagnosed cases (at 32, 34, 48, 53m) all with DS-TB who did not have baseline PET/CT consistent with TB, were due to infection after baseline (appendix pp 13, 20, 21). However, notably, all were QFT+ at baseline, so whether the distantly diagnosed TB episode was due to infection present at baseline or a subsequent infection cannot be determined. Regardless, the other 10/14 (71%) bacteriologically confirmed cases that occurred in baseline QFT-positive individuals all had baseline PET/CT-TB: six diagnosed at baseline and four on follow-up, within 36m.

To investigate potential index linkage in more detail, we focused on the 14/18 culture positive cases to compare full drug sensitivity profiles. There was only one additional Xpert-positive RR-TB case for whom culture wasn't available. This participant was QFT-negative at baseline with PET/CT-other lung lesions. Their index had RIF and INH resistance and so their linkage is possible but cannot be confirmed. The four untreated Xpert-positive culture-negative QFT+ asymptomatic participants (two baseline, two follow up) were all Xpert RIF-sensitive, two previously treated for DS-TB, 2-8 yrs prior.

Of the 14 culture positive cases, seven (50%) were DR-TB, all with baseline PET/CT-TB (appendix pp 13). Of these, 6/7 (86%) (from three baseline and three follow-up sputa [8,32,34m]) were confirmed with drug resistance similar to the index case. Two were detected by bulk culture and 4 following selective culture on 7H10/RIF agar plates to identify minority resistant isolates. Three [taken at 0, 8 and 32m] had both index and contact strains available for WGS, confirming linkage, with < 10 SNP different. One of these baseline cases was initially diagnosed drug sensitive with the index-matching strain (5-8 SNP different) only being identified during follow-up on sputa taken at 8 and 13m following DS-TB treatment initiation, with the participant stopping treatment after 3 months, indicating a possible low level mixed strain infection that was missed at baseline and incorrect initial DS-TB diagnosis. The one DR-TB case that did not match the index case was confirmed by bulk WGS to have mixed infection at baseline: 97% sublineage 4.1.2.1 and 3% sublineage 4.3.2.1. A low abundance 4.3.2 strain was identified rifampicin-resistant following plating the bulk culture on 7H10/RIF and resistant colonies cultured for phenotypic and genotypic confirmation. Both strains did not match the index case that was XDR-TB and lineage 2.2AA1 (appendix pp 13). Of the remaining 3/10 (30%) culture-positive cases with baseline PET/CT-TB who were all DS-TB, one was previously diagnosed with DS-TB 8 years prior and did not complete treatment then. The other 4/14 culture-positive strains that were DS-TB were from those without baseline PET/CT consistent with TB, all diagnosed drug-sensitive on follow-up, and the one with paired WGS available [diagnosed at 32m] was confirmed WGS discordant.

Thus, in summary, of the 7/250 (3%) asymptomatic contacts diagnosed with DR-TB similar to the index case; 6/7 (86%) had baseline PET/CT-TB and 1/7 was baseline QFT-negative with other lung lesions.

Supplementary Tables

Demographics	PET/CT (250)	Excluded (261)	P-value
Age (years)	30 (23-43)	35 (28-49)	**<0.0001**
Sex (Male)	100 (40%)	79 (30.3%)	**0.026**
Previous TB	34 (13.6%)	78 (31.2%)	**<0.0001**
BCG scar present	82 (32.8%)%	103 (39.5%)	0.29
TB symptoms or signs*	0 (0%)	49 (18.8%)	**<0.0001**
HIV-1 infected*	0 (0%)	145 (58%)	**<0.0001**
Uncontrolled DM*	0 (0%)	20 (7.7%)	**<0.0001**
Pregnancy ≤3m*	0 (0%)	29 (11.1%)	**<0.0001**
BMI	28.2 (22.2-34)	26.6 (22.3-31.6)	0.091
Currently smoking	72 (28.8%)	58 (22.2%)	0.10
Ever smoked	85 (34%)	87 (33.3%)	0.93
Current alcohol use	149 (59.6%)	128/255 (50.2%)	**0.040**
Daily index contact	208 (83.2%)	223 (85.4%)	0.54

Table S1. Demographics of adult TB contacts who underwent and those excluded from, PET/CT. *Exclusion criteria. Values are n (%) for indicated variable category or median (IQR). Denominator values are indicated in the column descriptor or indicated in a cell when values were missing. Differences between categorical variables analysed by χ^2 or Fisher's exact test, numerical variables by Mann-Whitney test. Bold indicates a significant difference ($p<0.05$). BMI, body mass index; DM, Diabetes Mellites; pregnancy ≤3m, breastfeeding, pregnant or planning pregnancy within 3 months.

A All participants

Lesion type	Total participants	Total lesions<br><br>(av. number of lesions/ participant)	Total Infiltrates [%]<br><br>(number with cavities)	Total Scars [%]<br><br>(number with calcification)	Total Nodules [%]<br><br>(number with calcification)	Bronchiectasis	Other	Right lung<br>RB1<br>RB2<br>RB3<br>RB6<br>RBOther	Left lung<br>LB12<br><br>LB3<br>LB6<br>LBOther
**Subclinical TB**	**29**	**183**<br><br>(6.31)	**45** [24.6%]<br><br>(8)	**43** [23.5%]<br><br>(13)	**89** [48.6%]<br><br>(26)	**4**	**2**	**23** [12.6%]<br>**23** [12.6%]<br>**24** [13.1%]<br>**5** [2.7%]<br>**27** [14.8%]	**35** [19.1]<br><br>**12** [6.6%]<br>**9** [4.9%]<br>**25** [13.7%]
**Subclinical TB-inactive**	**30**	**151**<br><br>(5.03)	**1** [0.7%]<br><br>(0)	**63** [41.7%]<br><br>(24)	**79** [53.2]<br><br>(45)	**5**	**3**	**24** [15.9%]<br>**11** [7.3%]<br>**17** [11.5%]<br>**8** [5.3%]<br>**17** [11.3%]	**29** [19.2%]<br><br>**19** [12.6%]<br>**4** [2.6%]<br>**22** [14.5%]
**Other**	**83**	**139**<br><br>(1.67)	**5** [3.6%]<br><br>(0)	**4** [2.9%]<br><br>(0)	**118** [87.8%]	**2**	**10**	**11** [7.9%]<br>**17** [12.2%]<br>**14** [10.1%]<br>**4** [2.9%]<br>**39** [28.1%]	**9** [6.5%]<br><br>**6** [4.3%]<br>**7** [5.0%]<br>**32** [23.0%]
**Normal**	**108**	**0**<br><br>(0)	**0**	**0**	**0**	**0**	**0**	**0**	**0**
**TOTAL**	**250**	**473**<br><br>(1.89)	**51**<br><br>(8)	**110**<br><br>(37)	**286**	**11**	**15**		

B Participants with no previous TB diagnosis

Lesion type	Total participants	Total lesions<br><br>(av. number of lesions/ participant)	Total Infiltrates [%]<br><br>(number with cavities)	Total Scars [%]<br><br>(number with calcification)	Total Nodules [%]<br><br>(number with calcification)	Bronchiectasis	Other	Right lung<br>RB1<br>RB2<br>RB3<br>RB6<br>RBOther	Left lung<br>LB12<br><br>LB3<br>LB6<br>LBOther
**Subclinical TB**	**15**	**67**<br><br>(4.47)	**22** [32.8%]<br><br>(5)	**8** [11.9%]<br><br>(3)	**36** [53.7%]<br><br>(6)	**0**	**1**	**15** [22.4%]<br>**9** [13.4%]<br>**12** [17.9%]<br>**2** [3.0%]<br>**8** [11.9%]	**10** [14.9%]<br><br>**3** [4.5%]<br>**4** [6.0%]<br>**4** [6.0%]
**Subclinical TB-inactive**	**15**	**60**<br><br>(4)	**1** [0.7%]<br><br>(0)	**21** [35%]<br><br>(7)	**34** [53.2]<br><br>(20)	**3**	**1**	**10** [16.7%]<br>**4** [6.7%]<br>**7** [11.7%]<br>**3** [5.0%]<br>**7** [11.7%]	**12** [20.0%]<br><br>**3** [5.0%]<br>**1** [1.7%]<br>**13** [21.7%]
**Other**	**81**	**133**<br><br>(1.64)	**5** [3.6%]<br><br>(0)	**4** [3.0%]<br><br>(0)	**112** [84.2%]<br><br>(22)	**2**	**10**	**10** [7.5%]<br>**17** [12.8%]<br>**11** [8.3%]<br>**4** [3.0%]<br>**38** [28.6%]	**9** [6.8%]<br><br>**5** [3.8%]<br>**7** [5.3%]<br>**32** [24.0%]
**Normal**	**105**	**0**<br><br>(0)	**0**	**0**	**0**	**0**	**0**	**0**	**0**
**TOTAL**	**216**	**260**<br><br>(1.2)	**28**<br><br>(5)	**33**<br><br>(10)	**182**<br><br>(48)	**5**	**12**		

Table S2. Distribution of lesion types and spatial location (bronchopulmonary segment) by baseline PET/CT radiographic category.

RB = Right bronchopulmonary segments 1-6 and other, LB = Left Bronchopulmonary segment 1-6 and other.

	Variable	All HHC<br>No previous TB<br>n=216	PET/CT<br>TB<br>n=15	PET/CT<br>Inactive TB<br>n=15	PET/CT<br>Other lung lesions<br>n=81	PET/CT<br>Normal lung<br>n=105	p-value
DEMOGRAPHICS	Age (years)	28 (23-42.5)	**43 (28-52)**[1]	**47 (25-56)**[1]	29 (23-39)	27 (22-40)	**0.003**
	Sex, Males n (%)	83 (38%)	7 (47%)	4 (27%)	30 (37%)	42 (40%)	0.69
	Sex, Female n (%)	133 (62%)	8 (53%)	11 (73%)	51 (63%)	63 (60%)	
	Daily index contact, n (%)	180 (83%)	14 (93%)	12 (80%)	70 (86%)	84 (80%)	0.49
	>6hrs/day with index, n (%)	83/215 (39%)	6 (40%)	7 (47%)	36 (44%)	34/104 (33%)	0.37
	1st degree relative, n (%)	91 (42%)	4 (27%)	10 (67%)	39 (48%)	38 (36%)	0.11
	Smoking history, n (%)	66 (31%)	5 (33%)	5 (33%)	22 (27%)	34 (32%)	0.87
	BMI	28.53 (22.63-34.71)	28.18 (19.57-32.44)	29.03 (22.17-37.02)	29.27 (22.37-33.49)	28.36 (23.48-35.24)	0.60
CLINICAL INVESTIGATIONS	CXR TB suggestive (human), n (%)	18/215 (8.4%)	**7 (47%)**	**1/14 (7%)**	**2 (3%)**	**8 (8%)**	**<0.0001**
	CAD4TBv7 score	6.0 (3.31-14.74)	**32.23 (3.45-64.11)**[1]	5.64 (4.13-26.07)	6.20 (2.90-15.07)	5.24 (3.22-9.867)	**0.030**
	qXRv3 score	0.0088 (0.0056-0.016)	**0.21 (0.0068-0.73)**[1,2]	**0.018 (0.0086-0.038)**[1,2]	0.0081 (0.0051-0.014)	0.0085 (0.0056-0.014)	**0.0006**
	Lunitv3 score	1.120 (0.79-2.03)	**11.02 (1.00-96.21)**[1,2]	3.070 (0.79-5.08)	1.080 (0.80-1.83)	1.070 (0.75-1.69)	**0.0010**
	Proportion QFN+ Any , n (%)	171/214 (80%)	14 (93%)	12 (80%)	70 (86%)	75/103 (73%)	0.07
	QFT Nil IFNγ (IU/ml)	0.03 (0-0.13)	0.08 (0.01-0.15)	0 (0-0.08)	0.03 (0-0.18)	0.03 (0-0.12)	0.26
	QFT-Gold Ag-Nil IFNγ (IU/ml)	5.44 (0.5-27.45)	6.98 (1.05-20.53)	6.86 (0.92-19.44)	7.63 (0.93-48.4)	3.94 (0.13-19.17)	0.11
	QFT-Plus-1 Ag-Nil IFNγ (IU/ml)	4.78 (0.33-26.45)	6.89 (1.02-18.96)	10.7 (0.98-22.92)	5.53 (0.95-40.26)	2.68 (0.13-16.57)	0.13
	QFT-Plus-2 Ag-Nil IFNγ (IU/ml)	5.08 (0.44-29.86)	12.61 (1.16-24.88)	7.37 (0.77-20.15)	6.49 (0.76-48.44)	3.46 (0.23-20.5)	0.13
	CRP (mg/L)	3 (1-6)	4 (1.2-11)	5 (3-8)	2.3 (1-5)	2 (1-6)	0.05
	CRP ≥ 10mg/L, n (%)	32/213 (15.0%	4 (27%)	3 (20%)	11/80 (14%)	14/103 (14%)	0.54
	ESR (mm/Hr)	14 (5-29)	20 (8-33)	**27 (20-45)**[1,2]	10.5 (4-30)	13 (3-25)	**0.002**
	White cell count ($x10^9$/L)	5.9 (4.73-7.54)	7.56 (6.68-8.32)	5.33 (4.95-7.18)	5.78 (4.53-7.37)	5.79 (4.74-7.54)	0.06
	Neutrophils ($x10^9$/L)	3.17 (2.21-4.54)	4.8 (3.69-5.63)	2.6 (2.1-4.48)	2.94 (2.17-4.6)	3.16 (2.25-4.36)	**0.05**
	N:L	1.55 (1.1-2.22)	2.54 (1.48-3.2)	1.46 (1.1-2.37)	1.5 (1.09-2.21)	1.59 (1.07-2.04)	0.07
	N:M	8.0 (6.22-10.38)	10.62 (7.24-12.31)	7.21 (5.68-8.97)	8.02 (6.44-10.33)	7.97 (6.13-9.79)	0.12
	L:M	5.11 (4.05-6.26)	4.05 (3.47-5.74)	5.63 (3.44-6.55)	4.99 (4.31-6.73)	5.17 (4.06-6.26)	0.20
PET/CT IMAGING FINDINGS	Lung total lesion number	1 (0-2)	**5 (2-6)**[1]	3 (1-7)	1 (1-2)	0 (0-0)	**<0.0001**
	total infiltrates	0 (0-0)	**1 (1-2)**[2,3]	0 (0-0)	0 (0-0)	NA	**<0.0001**
	total fibrotic scars	0 (0-1)	**0 (0-2)**[2,3]	**1 (1-2)**[2]	0 (0-0)	NA	**<0.0001**
	total nodules	1 (0-3)	3 (0-4)	1 (0-3)	1 (1-2)	NA	0.31
	total cavities (range)	0 (0-2)	**0 (0-2)**[2,3]	0 (0-0)	0 (0-0)	NA	**<0.0001**
	Lung largest lesion size (mm)	6.69 (4.44-21)	**36.9 (12.9-56.3)**[2]	**30 (19.8-40.6)**[2]	5.27 (4-9.84)	NA	**<0.0001**
	FDG-avid Lung present (%)	32 (14.8%)	**14 (93.3%)**	**5 (33.3%)**	**13 (16.0%)**	**0 (0%)**	**<0.0001**
	Lung maximum VS	0 (0-1)	**3 (1-3)**[2,3]	0 (0-1)	0 (0-0)	NA	**<0.0001**
	Lung SUVmax	1.23 (0.83-1.69)	**5.21 (1.64-6.25)**[2]	**1.69 (1.28-1.96)**[2]	1.08 (0.8-1.36)	NA	**<0.0001**
	Lung HUmax	48 (-56-273)	**120 (41-481)**[2]	**555 (36-1190)**[2]	13 (-134.5-181.5)	NA	**0.003**
	LN total lesions	0 (0-2)	**2 (2-3)**[1,2]	**2 (0-5)**[1,2]	0 (0-2)	0 (0-1)	**<0.0001**
	FDG-avid LN present (%)	42 (19.4%)	**10 (66.7%)**	**8 (53.3%)**	**15 (18.5%)**	**9 (8.6%)**	**<0.0001**
	Largest abnormal LN (mm)	8.4 (5.42-11)	**12 (10.2-14.9)** [1,2,3]	8 (5.4-10.6)	8.2 (5-10.3)	7.8 (5.1-9.8)	**0.01**
	LN maximum VS	1 (1-3)	**3 (2.5-3)**[1,2]	2 (1-3)	1 (1-3)	1 (1-2)	**0.02**
	LN SUVmax	2.5 (1.9-4.16)	**4.68 (3.24-6.36)**[1,2]	2.96 (2.22-5.07)	2.75 (1.6-3.47)	2.11 (1.84-2.8)	**0.005**
	LN HUmax	612.50 (157-1096.50)	391 (168-1106)	797 (158-1244)	488 (136-1013)	669.5 (252-1159)	0.65
TB OUTCOMES	Spontaneous Sputum Xpert positive at baseline (treated)	0	0	0	0	0	-
	Bact. confirmed baseline	5 (2.3%)	**5 (33.3%)**	**0**	**0**	**0**	**<0.0001**
	Bact. confirmed follow-up	8/215 (3.7%)	**3 (20%)**	**1 (6.7%)**	**2 (2.5%)**	**2/104 (1.9%)**	**0.017**
	Clinically diagnosed follow-up	1/215 (0.47%)	1 (6.7%)	0	0	0/104	0.14
	Xpert pos only – not treated	2 (0.93%)	1[5] (6.7%)	0	0	1[4] (0.95%)	0.099
	Any treated TB	14 (6.5%)	**9 (60.0%)**	**1 (6.7%)**	**2 (2.5%)**	**2 (1.9%)**	**<0.0001**
	Any treated TB or Xpert pos only	16 (7.4%)	**10 (66.7%)**	**1 (6.7%)**	**2 (2.5%)**	**3 (2.9%)**	**<0.0001**
	Symptoms at TB treatment	5/14 (35.7%)	**1/9 (11.1%)**	**1/1 (100%)**	**2/2 (100%)**	**1/2 (50%)**	**0.023**
	Symptoms at culture positive TB	3/12 (25%)	**0/8 (0%)**	**1/1 (100%)**	**1/1 (100%)**	**1/2 (50%)**	**0.018**

Table S3. Baseline characteristics, clinical and radiographic findings, and TB outcomes of household TB contacts who underwent PET/CT with no previous TB diagnosis.

Values are n (%) or median (IQR). Denominator values are indicated in the column descriptor or indicated in a cell when values were missing. Relationship between PET/CT categories and baseline characteristics analysed for categorical variables by χ^2 or Fisher's exact test and numerical variables by Kruskal Wallis with *post hoc* analysis using Dunn's multiple comparison testing. Bold and superscript number indicates which *post hoc* numerical comparisons are significantly different ($p<0.05$): 1 = in comparison with no lung lesions, 2 = in comparison with other lung lesions, 3 = in comparison with Inactive-Subclinical TB. BMI, body mass index; Clinical TB, TB symptom positive *Mtb* culture and Xpert negative; CRP, C-reactive protein; CXR, chest X-ray; ESR, erythrocyte sedimentation rate; HU_{max}, maximum Hounsfield units; IFNγ, interferon-gamma; LN, lymph node; L:M, blood lymphocyte:monocyte ratio; N:L, blood neutrophil:lymphocyte ratio; N:M, blood neutrophil:monocyte ratio; QFT+, any QuantiFERON positive result; SUV_{max}, maximum standardised uptake value; VS, visual score; Xpert, GeneXpert version 3.0: superscript 4 = detected at baseline, 5 = detected at follow-up.

Baseline PET/CT	Months to TB	Baseline QFT	Previous TB ended years prior	Sx at TB	HIV at TB	Index Extended DST	HHC Extended DST	HHC SNPs to index	Linkage	Index cohabit years
**Treated Culture Positive**										
TB	0	+	-	ASx	-	RIF AMI(L) ETHAM(L) INH KAN(L) OFLX PZA	DS (bulk)	NA	DST not linked (WGS NA)	NC
TB	0	+	-	ASx	-	RIF RBU	DS (bulk) at baseline diagnosis; 8-13m follow-up (3m Tx DNC) RIF PZA (RR colony)	740; 5-8	Baseline not linked 8-13m (Post-Tx) linked WGS SNPs + DR	5
TB	0	+	-	ASx	-	RIF INH CYCLO ETHAM(L) RBU PZA	RIF INH PZA (bulk) RIF INH ETHAM(L) RBU PZA (RR colony)	1-3	WGS SNPs + MDR linked	9
TB	0	+	-	ASx	-	RIF	DS (bulk) RIF RBU (RR colony)	NA	DST linked (WGS NA)	NC
TB	0	+	-	ASx	-	RIF INH(L) ETHAM(L) PZA	DS (bulk)	NA	DST not linked (WGS NA)	3
TB	0	+	7 yrs (DS DNC)	ASx	-	RIF (Xpert), DS (culture)	DS (bulk) RIF (RR colony)	NA	DST linked (no index WGS)	NC
TB	1	+	-	ASx	-	RIF INH AMI OFLX lineage 2.2AA1	DS (bulk), WGS mixed strains: sublineages 97% 4.1.2.1, 3% 4.3.2.1 RIF RBU (RR colony), lineage 4.3.2	1172 - 1181	WGS SNPs + DST not linked	NC*
TB	11	+	8 yrs (DS DNC)	ASx	-	RIF INH	DS (bulk)	NA	DST not linked (no index WGS)	NC
TB	32	+	-	ASx	-	RIF INH(L) STREP(L) PZA RBU	RIF INH(L) STREP ETHIO (bulk)	2-3	WGS SNPs + MDR linked	31
TB	34	+	-	ASx	-	RIF (Xpert)	DS (bulk) RIF (RR colony)	NA	DST linked (no index WGS)	0.25
Normal lung	5	-	-	Sx	-	RIF INH STREP PZA	DS (bulk)	NA	DST not linked (WGS NA)	10
Normal lung	32	+	-	ASx	-	RIF INH STREP	DS (bulk)	780	WGS SNPs + DS not linked	NC
Inactive TB	48	+	-	Sx	-	RIF INH AMI OFLX	DS (bulk)	NA	DST not linked (WGS NA)	NC*
Other lung lesions	53	+	-	Sx	+	RIF INH	DS (bulk)	NA	DST not linked (WGS NA)	NC
**Treated Xpert Positive Culture Negative/NA**										
Other lung lesions	13	-	-	Sx	-	RIF INH	RIF (Xpert)	NA	DST potential linkage	NC
Inactive TB	34	+	9 yrs (DS TC)	Sx	+	RIF (Xpert)	DS (Xpert)	NA	DST not linked (WGS NA)	NC
**Untreated Xpert Positive Culture Negative**										
Normal lung	0	+	0	ASx	-	RIF (Xpert), DS (culture)	DS (Xpert)	NA	Linkage unconfirmable	26
Inactive TB	0	+	8 yrs (DS TC)	ASx	-	RIF (Xpert), DS (culture)	DS (Xpert)	NA	Linkage unconfirmable	21
TB	23	+	0	ASx	-	RIF INH AMI OFLX	DS (Xpert)	NA	DST not linked (WGS NA)	NC
Inactive TB	24	+	2 & 5 yrs (DS TC)	ASx	-	RIF INH AMI OFLX	DS (Xpert)	NA	DST not linked (WGS NA)	NC

Table S4. Microbiological and clinical characteristics of participants with culture positive TB or only Xpert positive samples, compared to their index case drug sensitivity profile.
*Participants co-habited together but not with index case. ASx, asymptomatic; bulk, bulk culture; DS, drug sensitive; DNC, did not complete treatment; DST, drug sensitivity testing; DR drug resistant; HHC, household contact; Index, TB index case of contact; L, low level resistant; Linkage, whether index case and HHC are linked via DST or whole genome sequence (WGS) via single nucleotide polymorphism (SNP) distance (<10 SNPs) or DR or MDR (multi-drug resistant) SNP pattern, red indicates linked; NA, not available; NC, not co-habiting with index; RR, rifampicin resistant; Sx, symptomatic. TC, treatment completed. AMI, amikacin; CAP, capreomycin; CYCLO, cyclosporin; ETHAM, ethambutol; ETHIO, ethionamide; INH, isoniazid; KAN, kanamycin; LEV, levofloxacin; MXF, moxifloxacin; OLFX, ofloxacin; PZA, pyrazinamide; RBU, rifabutin; RIF, rifampicin; STREP, streptomycin.

Variable	Category	Univariate		Multivariate model adjusting for previous TB		Multivariate model adjusting for previous TB, age and sex	
		HR (95% CI)	p value	aHR (95% CI)	p value	aHR (95% CI)	p value
**PRIMARY ANALYSIS: Outcome = All treated cases (n=18)**							
PET/CT n=250	PET/CT - Normal	REF		REF		REF	
	PET/CT - Other	1.30 (0.18-9.23)	0.79	1.30 (0.18-9.21)	0.80	1.36 (0.19-9.68)	0.76
	PET/CT - Inactive	3.55 (0.50-25.21)	0.21	5.23 (0.72-37.81)	0.10	7.11 (0.95-53.22)	0.056
	PET/CT - TB	**28.54 (6.37-127.81)**	**<0.0001**	**44.93 (9.66-209.07)**	**<0.0001**	**66.15 (12.92-338.74)**	**<0.0001**
Previous TB history n=250	No	REF		REF		REF	
	Yes	1.84 (0.61-5.60)	0.28	0.33 (0.10-1.05)	0.059	**0.31 (0.09 – 0.99)**	**0.049**
Age n=250	Per year	1.02 (0.99-1.06)	0.27			0.97 (0.93-1.01)	0.16
Sex n=250	Female	REF				REF	
	Male	1.55 (0.60-3.84)	0.36			1.33 (0.52-3.41)	0.55
Hours of contact n=248	≤6hours/day	REF					
	>6 hours/day	1.62 (0.64-4.07)	0.31				
QuantiFERON n=248	Negative	REF					
	Positive	1.68 (0.39-7.32)	0.49				
**SENSITIVITY ANALYSIS 1: Outcome = Culture positive cases only (n=14)**							
PET/CT n=250	PET/CT - Normal	REF		REF		REF	
	PET/CT - Other	0.65 (0.06-7.17)	0.73	0.65 (0.06-7.15)	0.72	0.69 (0.06-7.61)	0.76
	PET/CT - Inactive	1.78 (0.16-19.59)	0.64	2.90 (0.26-32.38)	0.39	4.40 (0.39-50.34)	0.23
	**PET/CT - TB**	**22.98 (5.02-105.19)**	**<0.0001**	**40.81 (8.65-192.42)**	**<0.0001**	**70.37 (13.50-366.91)**	**<0.0001**
Previous TB history n=250	No	REF		REF		REF	
	Yes	1.07 (0.24-4.80)	0.93	**0.19 (0.04-0.87)**	**0.032**	**0.19 (0.04-0.89)**	**0.035**
Age n=250	Per year	1.01 (0.97-1.05)	0.77			0.96 (0.91-1.00)	0.067
Sex n=250	Female	REF				REF	
	Male	1.15 (0.40-3.32)	0.79			1.07 (0.36-3.16)	0.90
**SENSITIVITY ANALYSIS 2: Outcome = All treated cases and untreated Xpert+ Culture- (n=22)**							
PET/CT n=250	PET/CT - Normal	REF		REF		REF	
	PET/CT - Other	0.86 (0.14-5.16)	0.87	0.86 (0.14-5.14)	0.87	0.89 (0.15-5.35)	0.90
	PET/CT - Inactive	**4.88 (1.09-21.80)**	**0.038**	**6.50 (1.42-29.82)**	**0.016**	**8.20 (1.72-39.11)**	**0.008**
	PET/CT - TB	**20.59 (5.85-72.43)**	**<0.0001**	**29.40 (7.93-109.02)**	**<0.0001**	**37.37 (9.28-150.51)**	**<0.0001**
Previous TB history n=250	No	REF		REF		REF	
	Yes	2.48 (0.97-6.33)	0.058	0.45 (0.17-1.23)	0.12	0.42 (0.16-1.16)	0.10
Age n=250	Per year	1.02 (0.99-1.05)	0.17			0.98 (0.95-1.02)	0.27
Sex n=250	Female	REF				REF	
	Male	1.88 (0.81-4.35)	0.14			1.52 (0.65-3.57)	0.33
**SENSITIVITY ANALYSIS 3: Outcome = All treated cases if QFT+ at baseline (n=16)**							
PET/CT n=205	PET/CT - Normal	REF		REF		REF	
	PET/CT - Other	1.07 (0.07-17.06)	0.96	1.06 (0.07-16.92)	0.97	1.11 (0.07-17.72)	0.94
	PET/CT - Inactive	5.67 (0.51-62.42)	0.16	8.97 (0.80-100.67)	0.075	**15.24 (1.25-185.13)**	**0.033**
	PET/CT - TB	**43.14 (5.60-332.56)**	**<0.0001**	**71.94 (9.06-571.07)**	**<0.0001**	**109.70 (12.81-939.26)**	**<0.0001**
Previous TB history n=205	No	REF		REF		REF	
	Yes	1.71 (0.55-5.29)	0.36	**0.29 (0.09-0.94)**	**0.039**	**0.23 (0.07-0.78)**	**0.018**
Age n=205	Per year	1.02 (0.98-1.06)	0.29			0.97 (0.93-1.01)	0.16
Sex n=205	Female	REF				REF	
	Male	2.10 (0.78-5.65)	0.14			2.12 (0.76-5.92)	0.15

Table S5. Univariate and multivariate analyses of the risk to develop TB over the study periods due to baseline PET/CT lung parenchymal category and main covariates.

Cox proportional hazards regression was used to estimate hazard ratios (HRs) and 95% confidence intervals (CIs) for factors associated with TB diagnosis. Time zero was defined as day of first PET/CT, and participants were

censored at the earliest of study end, loss to follow-up (last time known TB-free), or non-TB death. Univariate analyses were first performed. Variables included in the multivariate model were selected based of univariate significance, clinical relevance and prior evidence:

Primary analysis: 18 cases; 14 culture positive + 4 additional treated symptomatic TB (2 clinically diagnosed and 2 Xpert positive – 1 culture negative and 1 culture unavailable).

Sensitivity analysis 1: 14 culture positive.

Sensitivity analysis 2: 22 cases; 14 culture positive + 4 additional treated symptomatic TB + 4 asymptomatic cases with positive Xpert (Xpert+) and negative culture (culture-).

Sensitivity analysis 3: 16 cases; restricted to baseline QuantiFERON-TB positive (QFT+, on QFT-Gold or QFT-Plus) individuals. 13 culture positive + 3 additional treated symptomatic TB (2 clinically diagnosed and 1 Xpert positive culture negative.

	Variable	PET/CT-TB or Inactive TB<br>No previous TB,<br>TB treated<br>n=10	PET/CT-TB or Inactive TB<br>No previous TB,<br>No TB treated<br>n=20[¶]	p-value
DEMOGRAPHICS	Age (years)	34.5 (28-48)	48 (28-55.5)	0.21
	Sex, Male n (%)	6 (60%)	5 (25%)	0.11
	Sex, Female n (%)	4 (40%)	15 (75%)	
	Daily contact with index (%)	9 (90%)	17 (85%)	0.70
	Smoking history, n (%)	5 (50%)	5 (25%)	0.17
	BMI	**20.26 (19.57-29.26)**	**30.97 (25.57-37.13)**	**0.030**
CLINICAL INVESTIGATIONS	CXR TB suggestive (medical officer), n (%)	7 (70%)	1/19 (5.3%)	**<0.0001**
	CAD4TBv7 score	**46.43 (29.78-65.60)**	**4.24 (3.45-19.10)**	**<0.0001**
	qXRv3 score	**0.43 (0.17-0.82)**	**0.012 (0.0068-0.031)**	**<0.0001**
	Lunitv3 score	**79.42 (9.56-97.62)**	**1.030 (0.84-3.64)**	**<0.0001**
	Proportion QFN+ (%) Any	10 (100%)	16 (80%)	0.13
	QFT Nil IFNγ (IU/ml)	**0.13 (0.07-0.23)**	**0 (0-0.07)**	**0.007**
	QFT-Gold Ag-Nil IFNγ (IU/ml)	6.97 (3.11-20.53)	8.01 (0.98-17.81)	0.64
	QFT-Plus-1 Ag-Nil IFNγ (IU/ml)	6.64 (2.39-18.96)	12.01 (0.98-22.26)	0.72
	QFT-Plus-2 Ag-Nil IFNγ (IU/ml)	13.89 (2.58-60.57)	7.88 (0.77-20.15)	0.30
	CRP (mg/L)	3.5 (2-9)	5 (3-10.5)	0.61
	CRP ≥ 10 mg/L	2 (20%)	5 (25%)	0.76
	ESR (mm/Hr)	29 (20-40)	24.5 (15.5-40.5)	0.78
	White cell count (x10^9/L)	**7.82 (6.92-8.32)**	**5.84 (5.05-7.3)**	**0.020**
	Neutrophil count (x10^9/L)	**5.09 (4.15-5.63)**	**3.47 (2.1-4.51)**	**0.016**
	Monocyte count (x10^9/L)	0.46 (0.41-0.54)	0.39 (0.30-0.39)	0.16
	Lymphocyte count (x10^9/L)	1.9 (1.63-2.28)	1.90 (1.64-2.40)	0.96
	N:L	2.6 (1.86-3.06)	1.51 (1.03-2.42)	0.10
	N:M	**10.72 (8.36-12.55)**	**7.24 (5.89-8.97)**	**0.053**
	L:M	4.28 (3.47-5.72)	5.19 (3.46-6.36)	0.35
PET/CT IMAGING FINDINGS	PET/CT-TB	**9 (90%)**	**6 (30%)**	**0.0052**
	Lung total lesion number	**6 (5-8)**	**2 (1-6)**	**0.023**
	total infiltrates (IQR)	**1.5 (0-3)**	**0 (0-0)**	**0.0016**
	total fibrotic scars (IQR)	1 (0-2)	1 (0.5-2)	0.83
	total nodules (IQR)	3 (0.75-4.25)	1 (0-3)	0.20
	total cavities (IQR)	**0 (0-1.25)**	**0 (0-0)**	**0.030**
	≥ 1 Cavity present	**3 (30%)**	**0 (0%)**	**0.030**
	Lung largest lesion size (mm)	43.15 (19.8-56.3)	28.35 (14.25-42.39)	0.18
	Lung HUmax	218.5 (120-782)	131 (16.5-725.5)	0.39
	Lung maximum VS	**3 (3-3)**	**0 (0-1)**	**<0.0001**
	Lung SUVmax	**5.94 (5.21-8.96)**	**1.55 (1.22-1.92)**	**<0.0001**
	Proportion with FDG-avid LN	6 (60%)	12 (60%)	1.00
	Largest abnormal LN (mm)	12 (10.2-15)	8.98 (6.19-12.5)	0.088
	LN HUmax	1049 (158-1192)	627 (179-975)	0.72
	LN maximum VS	3 (2-3)	3 (1.5-3)	0.67
	LN SUVmax	4.81 (2.4-6.66)	3.58 (2.31-5.04)	0.49

[¶] Includes 1 Xpert-positive untreated, during follow-up.

Table S6. Characteristics of participants with PET/CT consistent with TB or inactive TB with no previous TB history in relation to 5-year TB diagnosis and treatment

Values are n (%) or median (IQR). Relationship between TB outcome and baseline characteristics analysed for categorical variables by Fisher's exact test, numerical variables by Mann-Whitney t test. Bold number indicates which comparisons are significantly different ($p \leq 0.05$). BMI, body mass index; CRP, C-reactive protein; CXR, chest X-ray; ESR, erythrocyte sedimentation rate; FDG, Fluoro deoxyglucose; HUmax, maximum Hounsfield units; IFNγ, interferon-gamma; LN, lymph node; N:L, neutrophil:lymphocyte ratio in blood; N:M, neutrophil:monocyte ratio in blood; L:M, lymphocyte:monocyte ratio in blood; QFT+, any QuantiFERON positive result; SUVmax, maximum standardised uptake value; VS, visual score.

	Variable	PET/CT-TB TB treated n=12	PET/CT-TB No TB[¶] n=17	p=value
DEMOGRAPHICS	Age (years)	37.0 (28.3-53.4)	46.0 (35.0-50.5)	0.21
	Sex, Males n (%)	7 (58%)	8 (47%)	0.71
	Sex, Female n (%)	5 (42%)	9 (53%)	
	Previous TB history	3 (25%)	11 (65%)	0.060
	Daily index contact, n (%)	10 (83%)	14 (82%)	1.0
	>6hrs/day with index, n (%)	6 (50%)	8 (47%)	1.0
	Smoking history, n (%)	6 (50%)	6 (35%)	0.47
	BMI	21.9 (19.6-29.3)	25.0 (21.5-31.0)	0.35
CLINICAL INVESTIGATIONS	CXR Suggestive of TB (medical officer), n (%)	8 (66.7%)	8 (47.1%)	0.45
	CAD4TBv7 score	56.16 (33.10-66.77)	19.10 (3.73-64.53)	0.088
	qXRv3 score	0.58 (0.21-0.84)	0.14 (0.0078-0.69)	0.14
	Lunitv3 score	83.52 (13.29-97.39)	22.21 (1.01-83.32)	0.064
	Proportion QFN+ Any, n (%)	12 (100.0%)	16 (94.1%)	1.0
	QFT Nil value IFNγ (IU/ml)	0.08 (0.02-0.18)	0.05 (0.00-0.13)	0.68
	QFT-Gold Ag-Nil IFNγ (IU/ml)	6.97 (2.02-14.94)	29.15 (6.57-71.05)	0.097
	QFT-Plus-1 Ag-Nil IFNγ (IU/ml)	6.64 (1.52-15.75)	22.26 (6.76-60.91)	0.12
	QFT-Plus-2 Ag-Nil IFNγ (IU/ml)	10.13 (1.56-20.57)	24.88 (5.28-72.40)	0.23
	CRP (mg/L)	4.50 (2.00-17.25)	3.00 (1.00-11.50)	0.30
	CRP ≥10 mg/L, n (%)	3 (25%)	6 (36%)	0.69
	ESR (mm/Hr)	**31.50 (20.25-44.25)**	**15.00 (5.50-24.50)**	**0.014**
	White cell count ($x10^9$/L)	**7.53 (6.98-8.28)**	**6.62 (5.03-7.86)**	**0.021**
	Neutrophils ($x10^9$/L)	**4.67 (3.91-5.58)**	**3.89 (2.65-4.55)**	**0.030**
	N:L	2.39 (1.88-2.98)	1.96 (1.25-2.41)	0.097
	N:M	10.63 (6.36-12.42)	8.40 (7.13-11.66)	0.59
	L:M	3.83 (2.60-5.42)	5.26 (3.56-6.92)	0.066
PET/CT IMAGING FINDINGS	Lung total parenchymal lesions	5.50 (5.00-8.75)	6.00 (2.50-10.00)	0.91
	total infiltrates	2.00 (0.25-2.75)	1.00 (0.00-2.00)	0.24
	total fibrotic scars	1.50 (0.00-2.00)	1.00 (0.00-3.00)	0.88
	total nodules	3.00 (0.25-4.75)	3.00 (1.00-5.50)	0.74
	total cavities	0 (0-1)	0 (0-0)	0.056
	≥ 1 Cavity present	5 (42%)	2 (12%)	0.092
	Lung largest lesion size (mm)	51.70 (21.38-60.95)	40.50 (22.80-56.30)	0.50
	Lung maximum VS	**3.00 (2.25-3.00)**	**2.00 (1.00-3.00)**	**0.024**
	Lung SUVmax	**5.94 (2.92-8.53)**	**2.25 (1.56-4.01)**	**0.0051**
	Lung HUmax	194 (92-904)	148 (64-632)	0.78
	LN total lesions	2.00 (0.25-3.75)	2.00 (0.00-3.00)	0.42
	Proportion with FDG-avid LN, n (%)	8 (67%)	8 (47%)	0.45
	Largest abnormal LN (mm)	11.80 (9.05-13.50)	10.10 (6.21-14.80)	0.55
	LN HU max	180 (82-1178)	278 (156-441)	1.0
	LN max VS	3.00 (2.00-3.00)	3.00 (1.00-3.00)	0.60
	LN max SUV	4.56 (2.65-6.36)	3.18 (2.43-3.71)	0.26

¶ Includes 1 Xpert-positive untreated, during follow-up.

Table S7. Characteristics of participants with PET/CT consistent with TB (including those with previous TB history) in relation to 5-year TB diagnosis and treatment.

Values are n (%) or median (IQR). Relationship between TB outcome and baseline characteristics analysed for categorical variables by Fisher's exact test, numerical variables by Mann-Whitney t test. Bold number indicates which comparisons are significantly different ($p \leq 0.05$). BMI, body mass index; CRP, C-reactive protein; CXR, chest X-ray; ESR, erythrocyte sedimentation rate; FDG, Fluoro deoxyglucose; HUmax, maximum Hounsfield units; IFNγ, interferon-gamma; LN, lymph node; N:L, neutrophil:lymphocyte ratio in blood; N:M, neutrophil:monocyte ratio in blood; L:M, lymphocyte:monocyte ratio in blood; QFT+, any QuantiFERON positive result; SUVmax, maximum standardised uptake value; VS, visual score.

Supplementary Figures

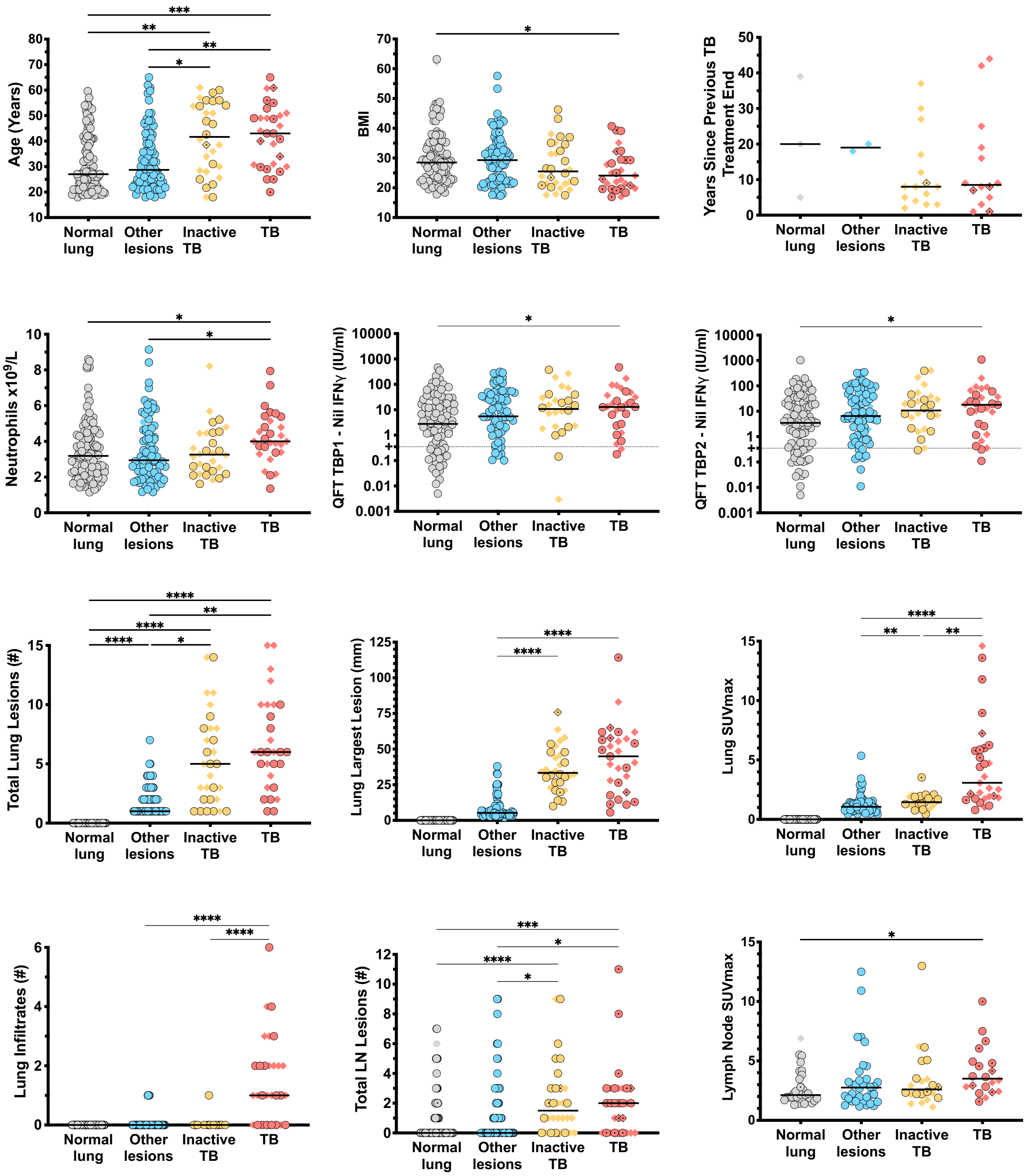

Figure S1. Demographic, blood, and radiographic findings by baseline PET/CT lung classification Diamond symbols indicate those with previous TB, circles without previous TB and internal dots indicate those TB diagnosed and treated during the study. Line indicates median. #, number; BMI, body mass index; IFNγ, interferon-gamma; LN, lymph node; Neut:Lymph, blood neutrophil:lymphocyte ratio; QFT TBP1, QuantiFERON-TB Plus tube 1, QFT TBP2, QuantiFERON-TB Plus tube 2; SUVmax, maximum standardised uptake value . Kruskal Wallis with post hoc analysis using Dunn's multiple comparisons test; *, p<0.05; **, p<0.01; ***, p<0.001, ****, p<0.0001.

Figure S2

A Baseline culture positive

Participant details | **Baseline PET/CT** | **Baseline CXR** | **Follow-up PET/CT**

Male 40y, QFT+	Asymptomatic, Culture positive, Baseline		
Previous TB	1:DS, 7 yrs prior, Tx did not complete		
Baseline CXR	Abnormal: Active TB		
Sputum 0m	Cul spont: 1/1<br>Cul induced:2/2	GX spont: 1/1<br>GX induced:2/2	SMR spont:0/1<br>SMR induced: 1/2
Resistance 0m	Cul: DS; RR colony: RIF		GX: NA
Index Resistance	Cul: DS		GX: RIF

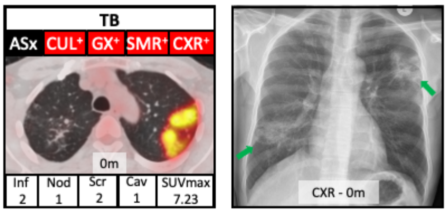

Male 20y, QFT+	Asymptomatic, Culture positive, Baseline		
Previous TB	0		
Baseline CXR	Abnormal: Active TB		
Sputum 0m	Cul spont: 1/1<br>Cul induced:2/2	GX spont: 0/1<br>GX induced:1/2	SMR spont:0/1<br>SMR induced: 1/2
Sputum 13m	Cul induced:1/1	GX induced:1/1	SMR induced: 1/1
Resistance 0m	Cul: DS		GX: DS
Resistance 13m	Cul: DS; RR colony: RIF PZA		GX: DS
Index Resistance	Cul: RIF RBU		GX: RIF

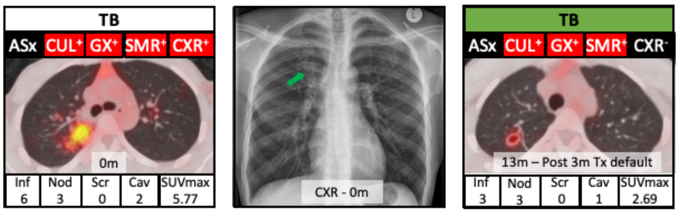

Female 29y, QFT+	Asymptomatic, Culture positive, Baseline		
Previous TB	0		
Baseline CXR	Abnormal: Active TB		
Sputum 0m	Cul spont: 0/1<br>Cul induced:1/2	GX spont: 0/1<br>GX induced:0/2	SMR spont:0/1<br>SMR induced: 0/2
Resistance	Cul: DS		GX: NA
Index Resistance	Cul: RIF INH AMI(L) ETHAM(L) KAN(L) OFLX PZA		GX: RIF

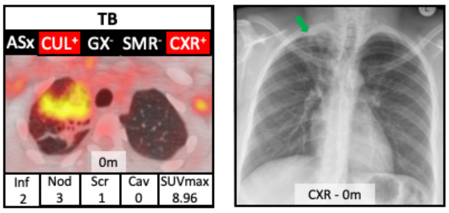

Male 25y, QFT+	Asymptomatic, Culture positive, Baseline		
Previous TB	0		
Baseline CXR	Abnormal: Active TB		
Sputum 0m	Cul induced:1/3	GX induced:0/3	SMR induced: 0/3
Resistance 0m	Cul: DS		GX: NA
Index Resistance	Cul: RIF INH(L) ETHAM(L) PZA		GX: RIF

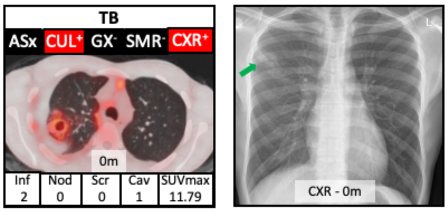

Female 29y, QFT+	Asymptomatic, Culture positive, Baseline		
Previous TB	0		
Baseline CXR	Abnormal: Active TB		
Sputum 0m	Cul induced:2/3	GX induced:0/3	SMR induced: 0/3
Resistance 0m	Cul: RIF INH PZA;<br>RR colony: RIF INH ETHAM(L) RBU PZA		GX: NA
Index Resistance	Cul: RIF INH CYCLO ETHAM(L) RBU PZA		GX: RIF

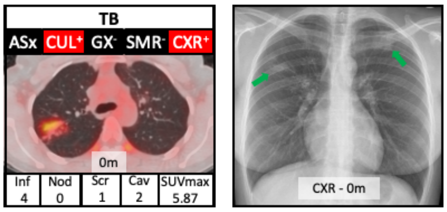

Female 28y, QFT+	Asymptomatic, Culture positive, Baseline		
Previous TB	0		
Baseline CXR	Normal		
Sputum 0m	Cul induced:1/3	GX induced:0/3	SMR induced: 0/3
Resistance 0m	Cul: DS;<br>RR colony: RIF RBU		GX: NA
Index Resistance	Cul: RIF (RBU not tested)		GX: RIF

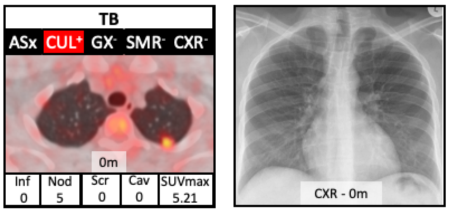

B Follow-up culture positive

Participant details

Male 55y, QFT+	Asymptomatic, Culture positive, Month 1		
Previous TB	0		
Baseline CXR	Abnormal: Active TB		
Sputum 0m	Cul spont: 0/1<br>Cul induced:0/2	GX spont:0/1<br>GX induced:0/2	SMR spont:0/1<br>SMR induced: 0/2
Sputum 1m	Cul induced: 1/1	GX induced: 0/1	SMR induced: 0/1
Resistance 1m	Cul: DS;<br>RR colony: RIF INH RBU		GX: NA
Index Resistance	Cul: RIF INH AMI OFLX		GX: RIF

Female 27y, QFT-	Symptomatic, Culture positive, Month 5		
Previous TB	0		
Baseline CXR	Abnormal: Old TB		
Sputum 0m	Cul spont: NA<br>Cul induced:0/3	GX spont: NA<br>GX induced:0/3	SMR spont: NA<br>SMR induced: 0/3
Sputum 5m	Cul induced: 1/1	GX induced: 1/1	SMR induced: ND
Resistance 5m	Cul: DS		GX: DS
Index Resistance	Cul: RIF INH STREP PZA		GX: RIF

Female 34y, QFT+	Asymptomatic, Culture positive, Month 11		
Previous TB	1:DS, 8 yrs prior, Tx did not complete		
Baseline CXR	Normal		
Sputum 0m	Cul spont: 0/1<br>Cul induced:0/2	GX spont: 0/1<br>GX induced:0/2	SMR spont:0/1<br>SMR induced: 0/2
Sputum 11m	Cul induced: 1/1	GX induced: 0/1	SMR induced: 0/1
Resistance 11m	Cul: DS		GX: NA
Index Resistance	Cul: RIF INH		GX: RIF

Male 40y, QFT+	Asymptomatic, Culture positive, Month 32		
Previous TB	0		
Baseline CXR	Normal		
Sputum 0m	Cul spont: 0/1<br>Cul induced:0/2	GX spont: 0/1<br>GX induced:0/2	SMR spont:0/1<br>SMR induced: 0/2
Sputum 6m	Cul induced: 0/1	GX induced: 0/1	SMR induced: 0/1
Sputum 32m	Cul spont: 1/1<br>Cul induced:3/3	GX spont: 1/1<br>GX induced:1/3	SMR spont: 0/1<br>SMR induced: 0/3
Resistance 32m	Cul: RIF INH(L) STREP ETHIO		GX: RIF
Index Resistance	Cul: RIF INH(L) STREP(L) PZA RBU		GX: RIF

Female 51y, QFT+	Asymptomatic, Culture positive, Month 32		
Previous TB	0		
Baseline CXR	Normal		
Sputum 0m	Cul spont: 0/1<br>Cul induced:0/2	GX spont: 0/1<br>GX induced:0/2	SMR spont:0/1<br>SMR induced: 0/2
Sputum 7m	Cul induced: 0/1	GX induced: 0/1	SMR induced: 0/1
Sputum 32m	Cul spont: 0/1<br>Cul induced:1/2	GX spont: 0/1<br>GX induced:0/2	SMR spont: 0/1<br>SMR induced: 0/2
Resistance 32m	Cul: DS		GX: NA
Index Resistance	Cul: RIF INH STREP		GX: RIF

Female 48y, QFT+	Asymptomatic, Culture positive, Month 34		
Previous TB	0		
Baseline CXR	Abnormal: Other		
Sputum 0m	Cul spont: 0/1<br>Cul induced:0/2	GX spont: 0/1<br>GX induced:0/2	SMR spont:0/1<br>SMR induced: 0/2
Sputum 12m	Cul induced: 0/1	GX induced: 0/1	SMR induced: 0/1
Sputum 34m	Cul induced: 2/2	GX induced: 1/2	SMR induced: 0/2
Resistance 34m	Cul: DS; RR colony = RIF		GX: DS
Index Resistance	Cul: NA		GX: RIF

Male 42y, QFT+	Symptomatic, Culture positive, Month 48		
Previous TB	0		
Baseline CXR	Abnormal: Old TB		
Sputum 0m	Cul spont: 0/1<br>Cul induced:0/2	GX spont: 0/1<br>GX induced:0/2	SMR spont:0/1<br>SMR induced: 0/2
Sputum 6m	Cul induced: 0/1	GX induced: 0/1	SMR induced: 0/1
Sputum 48m	Cul spont.: 1/2	GX spont.: 0/2	SMR spont.: 0/2
Resistance	Cul: DS		GX: NA
Index Resistance	Cul: RIF INH AMI OFLX		GX: RIF

Female 21y, QFT+	Symptomatic, Culture positive, Month 53, HIV+		
Previous TB	0		
Baseline CXR	Normal		
Sputum 0m	Cul spont: 0/1<br>Cul induced:0/2	GX spont: 0/1<br>GX induced:0/2	SMR spont:0/1<br>SMR induced: 0/2
Sputum 53m HIV+	Cul pleural Fluid: 1/1<br>Cul sputum: NA	GX pleural fluid: 0/1<br>GX sputum: 1/1	SMR pleural fluid: 0/1<br>SMR sputum: 0/1
Resistance	Cul: DS		GX: DS
Index Resistance	Cul: RIF INH		GX: RIF

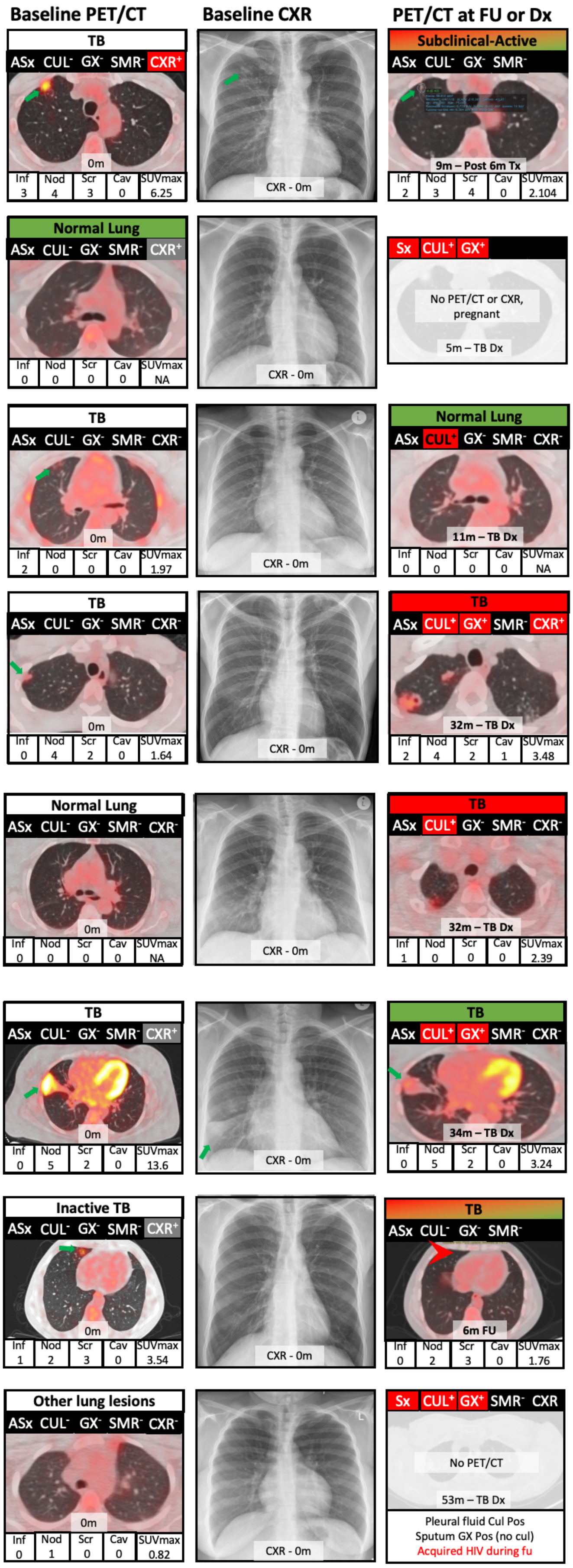

C Follow-up clinical diagnosis or Xpert-positive culture-negative or unavailable, received treatment

Participant details | **Baseline PET/CT** | **Baseline CXR** | **PET/CT on FU or Dx**

Female 29y, QFT-	Symptomatic, Xpert positive, Month 13		
Previous TB	0		
Baseline CXR	Normal		
Sputum 0 m	Cul spont: 0/1<br>Cul induced:0/2	GX spont: 0/1<br>GX induced:0/2	SMR spont:0/1<br>SMR induced: 0/2
Sputum 13 m	Cul spont: NA	GX spont: 1/1	SMR spont: NA
Resistance 13 m	Cul: NA		GX: RIF
Index Resistance	Cul: RIF INH		GX: RIF

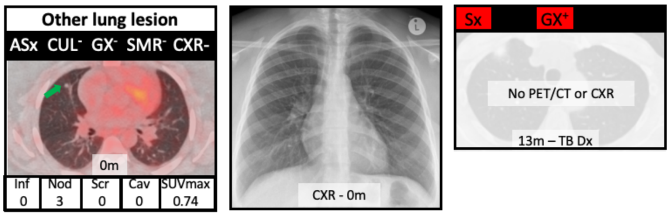

Male 60y, QFT+	Symptomatic, Clinical diagnosis, Month 24		
Previous TB	1: DS, 1 yr prior, completed treatment		
Baseline CXR	Abnormal: Active TB		
Sputum 0 m	Cul spont: 0/1<br>Cul induced:0/2	GX spont: 0/1<br>GX induced:0/2	SMR spont:0/1<br>SMR induced: 0/2
Sputum 9 m	Cul induced: 0/1	GX induced: 0/1	SMR induced: 0/1
BAL 11 m	RUL: 0/1	LUL: 0/1	
Sputum 16 m	Cul induced: 0/3	GX induced: 0/3	SMR induced: 0/3
Sputum 24 m	Cul induced: 0/5	GX induced: 0/5	SMR induced: 0/5
Resistance 24 m	Cul: NA – 6m DS treatment completed		GX: NA
Index Resistance	Cul: RIF PZA		GX: RIF

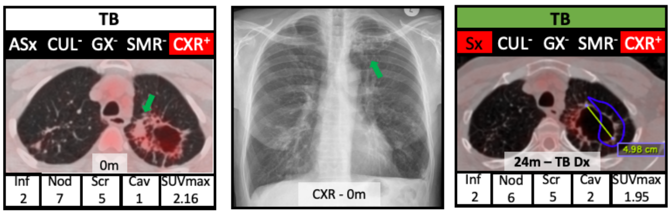

Male 35y, QFT+	Symptomatic, Xpert positive, Month 34, HIV+		
Previous TB	1:DS, 9 yrs prior, Tx completed		
Baseline CXR	Abnormal: Active TB		
Sputum 0m	Cul spont: 0/1<br>Cul induced:0/2	GX spont: 0/1<br>GX induced:0/2	SMR spont:0/1<br>SMR induced: 0/2
Sputum 34m HIV+	Cul induced: 0/3	GX induced: 1/3	SMR induced: 0/3
Resistance 34m	Cul: NA – 6m DS treatment completed		GX: DS
Index Resistance	Cul: NA		GX: RIF

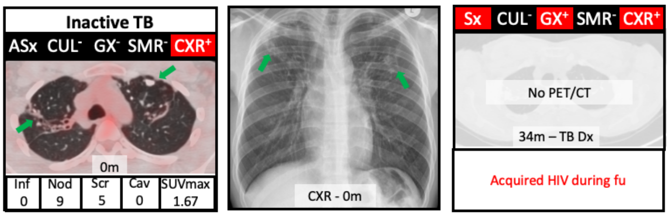

Male 56y, QFT+	Symptomatic, Clinical Diagnosis, Month 36		
Previous TB	0		
Baseline CXR	Abnormal: Active TB		
Sputum 0 m	Cul induced: 0/3	GX induced: 0/3	SMR induced: 0/3
Sputum 6 m	Cul induced: 0/1	GX induced: 0/1	SMR induced: 0/1
Sputum 24 m	Cul spont: 0/1<br>Cul induced:0/2	GX spont: 0/1<br>GX induced:0/2	SMR spont:0/1<br>SMR induced: 0/2
Sputum 36 m	Cul : NA	GX : NA	SMR : NA
Resistance 36 m	Cul: NA, treated LEVO LZD BDQ, outcome unknown		GX: NA
Index Resistance	Cul: RIF INH PZA RBU		GX: RIF

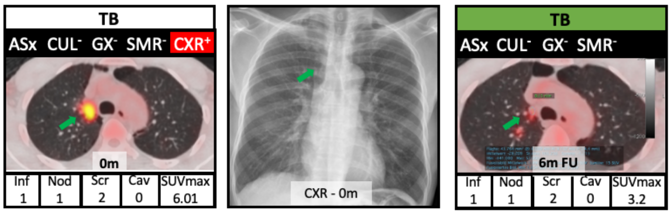

D Baseline and follow-up Xpert-positive culture-negative, no clinical diagnosis, no treatment

Participant details

Male 38y, QFT+	Asymptomatic, Xpert positive, Month 0		
Previous TB	1: DS, 2008, 8 yrs prior, treatment completed		
Baseline CXR	Normal		
Sputum 0m	Cul spont: 0/1<br>Cul induced:0/2	GX spont: 1/1<br>GX induced:0/2	SMR spont:0/1<br>SMR induced: 0/2
Sputum 11m	Cul induced: 0/1	GX induced: 0/1	SMR induced: 0/1
BAL 13m	Cul RUL: 0/1	Cul LUL: 0/1	
Resistance 0m	Cul: NA		GX: DS
Index Resistance	Cul: DS		GX: RIF

Male 27y, QFT+	Asymptomatic, Xpert positive, Month 0		
Previous TB	0		
Baseline CXR	Normal		
Sputum 0m	Cul spont: 0/1<br>Cul induced:0/2	GX spont: 0/1<br>GX induced:1/2	SMR spont:0/1<br>SMR induced: 0/2
Sputum 11m	Cul induced: 0/1	GX induced: 0/1	SMR induced: 0/1
Sputum 24m	Cul induced: 0/2	GX induced: 0/2	SMR induced: 0/2
Resistance 0m	Cul: NA		GX: DS
Index Resistance	Cul: DS		GX: RIF

Male 28y, QFT+	Asymptomatic, Xpert positive, Month 24		
Previous TB	2: DS, 5 yrs & 2 yrs prior, treatment completed		
Baseline CXR	Abnormal: Active TB		
Sputum 0m	Cul spont: 0/1<br>Cul induced:0/2	GX spont: 0/1<br>GX induced:0/2	SMR spont:0/1<br>SMR induced: 0/2
Sputum 12m	Cul induced: 0/3	GX induced: 0/3	SMR induced: 0/3
BAL 13m	Cul RLL: 0/1	Cul LLL: 0/1	
Sputum 24m	Cul spont: 0/1<br>Cul induced:0/2	GX spont: 1/1<br>GX induced:0/2	SMR spont:0/1<br>SMR induced: 0/2
Resistance 24m	Cul: NA		GX: DS
Index Resistance	Cul: RIF INH AMI OFLX		GX: RIF

Female 65y, QFT+	Asymptomatic, Xpert positive, Month 23		
Previous TB	0		
Baseline CXR	Normal		
Sputum 0 m	Cul induced: 0/3	GX induced: 0/3	SMR induced: 0/3
Sputum 10 m	Cul induced: 0/1	GX induced: 0/1	SMR induced: 0/1
Sputum 23 m	Cul induced:0/3	GX induced:1/3	SMR induced: 0/3
Resistance 23 m	Cul: NA		GX: DS
Index Resistance	Cul: RIF INH FLUOROQUINOLONES SECOND LINE INJECTABLES		GX: RIF

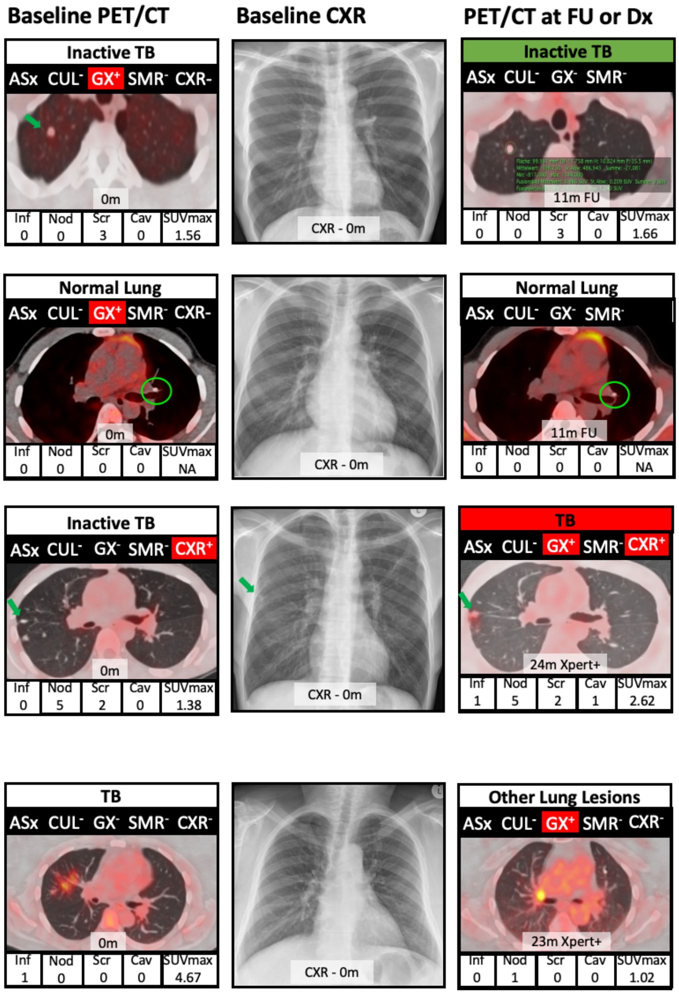

Figure S2. Radiographic and microbiological findings at baseline and TB diagnosis or follow-up. Shows details of all 22 participants diagnosed with TB by various definitions. For each, the table provides details of previous TB history, baseline chest X-ray (CXR) result and comprehensive microbiology of sputum and bronchoalveolar lavage (BAL, when performed), for each timepoint. The images show in the left column a single axial section from the baseline scan with (above image) detail of overall PET/CT category (top), details of symptoms (Sx, symptomatic; ASx, asymptomatic), positive (+) or negative (-) culture (CUL), Xpert (GX), and smear (SMR) and CXR result and (below image) number and nature of lesions (Inf, infiltrates; Nod, nodules; Scr, fibrotic scar; Cav, cavity; SUVmax, maximum parenchymal lesion standardized uptake value. The middle column shows the baseline CXR with lesions identified with green arrow. The right column shows single axial section at the repeat PET/CT 5-15m during follow-up (FU) or at the point of diagnosis (Dx) during follow-up if available. The background colour of the overall PET/CT category on the top of this image indicates if the lesions were improved (green), worsened (red) or mixed (green/red) compared to the prior PET/CT. HIV+ indicates two participants who seroconverted during follow-up.

A - baseline culture positive (n=6)
B - follow-up culture positive (n=8)
C - follow-up clinical diagnosis or Xpert positive culture negative/unavailable received treatment (n=4)
D – baseline or follow-up Xpert positive culture negative, no clinical diagnosis, no treatment (n=4).

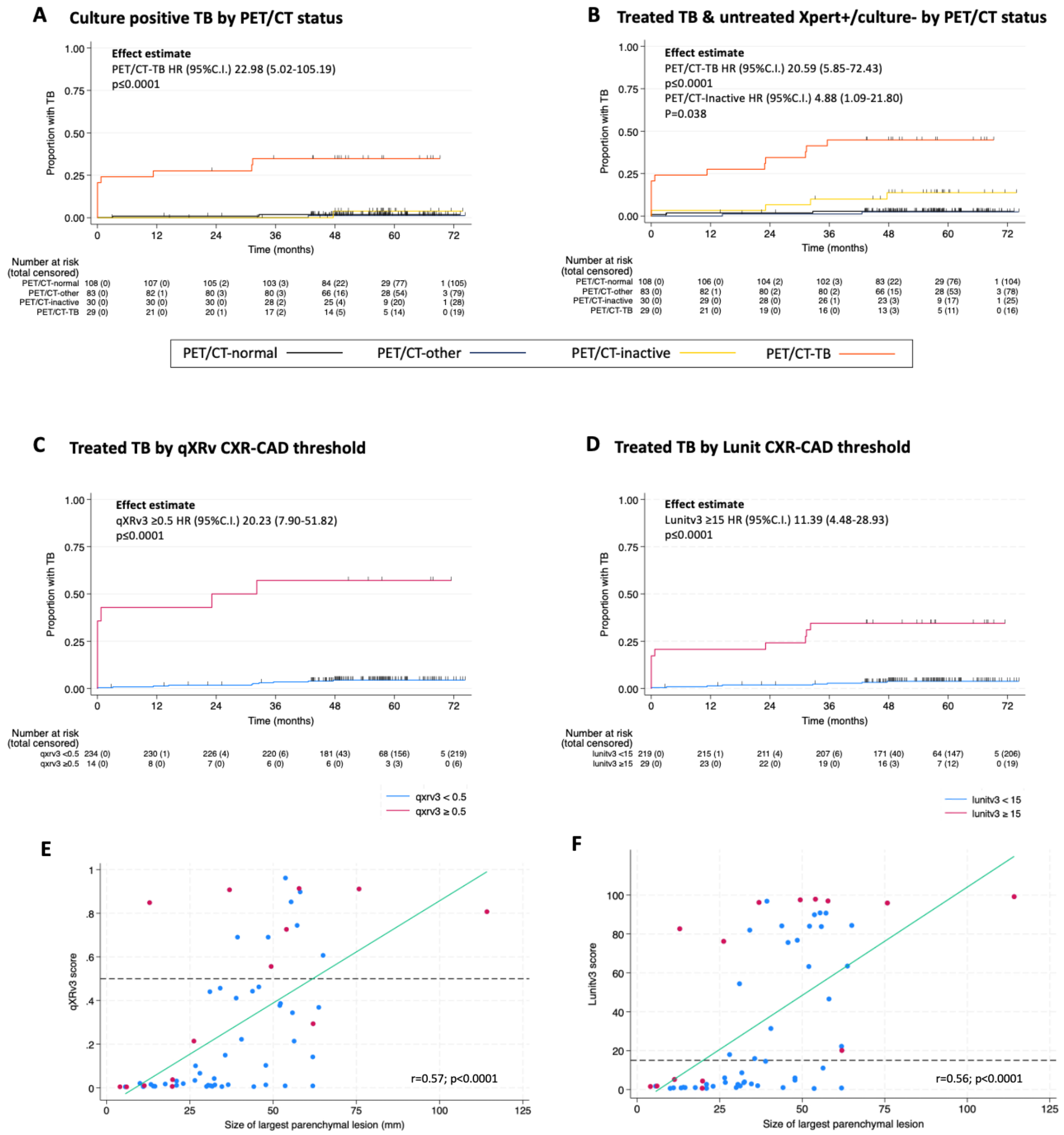

Figure S3. Baseline PET/CT lung category and chest radiograph computer-aided detection (CXR-CAD) scores in relation to TB outcome over study period and CXR-CAD scores in relation to size of largest baseline PET/CT parenchymal lesion.

(A-B) Kaplan-Meier failure plots showing proportion over study period of those (A) diagnosed and treated for culture positive TB or (B) proportion who received any TB treatment or had untreated Xpert-positive culture negative sputum by baseline PET/CT lung category (n=250). (C-D) Kaplan-Meier failure plots showing proportion over study period of those diagnosed and treatment for TB by CXR-CAD manufacturer's threshold suggestive of TB for baseline (C) qXR version 3.0.0 (qXRv3) and (D) Lunit INSIGHT CXR version 3.1.4.111 (Lunitv3) score for all participants with CXR-CAD (n=248). Hazard ratio (HR) for the specified case definition of TB over the median 4.7 year follow-up period. Plot time zero was defined as the date of first PET/CT or date of first CXR. The event was defined as the first confirmed diagnosis of tuberculosis (TB), based on either date of sample that resulted in positive microbiology that led to treatment or day of treatment initiated if microbiologically negative. Tick marks indicate participants censored at the earliest of: end of study follow-up, loss to follow-up (last date known TB free), or death. Scatter plot of (E) qXRv3 and (F) Lunitv3 score against length of largest parenchymal lesion for 58 participants with PET/CT-TB or PET/CT-inactive. The 14 participants subsequently bacteriologically confirmed at any point over follow-up are shown in red with the remainder in blue. The line of best fit is shown for all 58 participants. The same plots for CAD4TBv7 are shown in figure 3D and figure 5D.

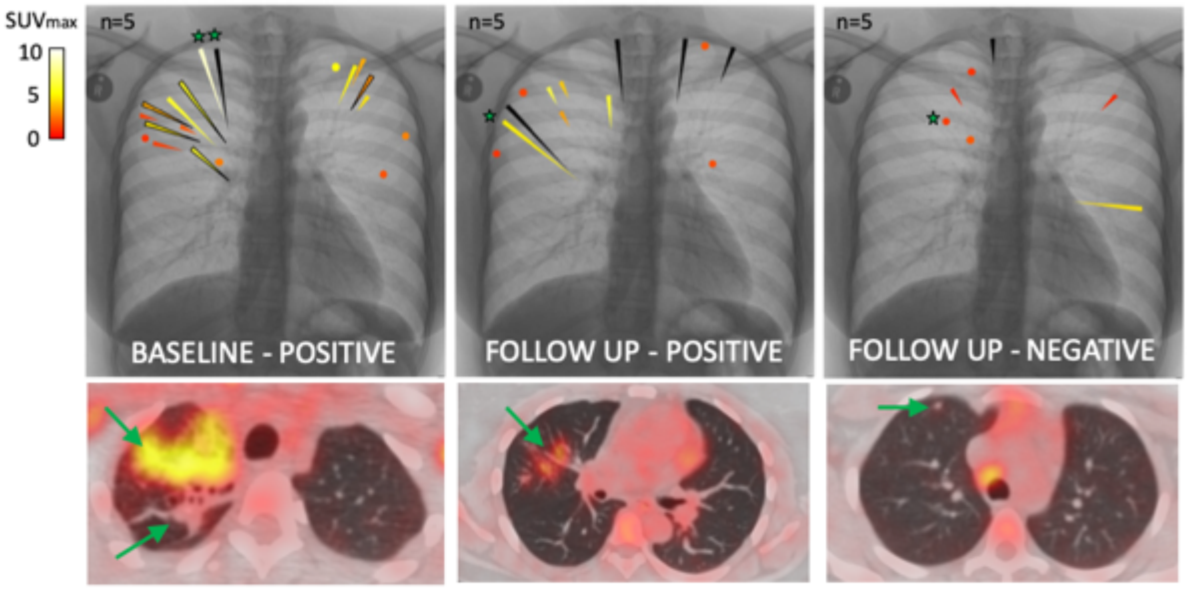

Figure S4. Distribution, size, type and metabolic activity of lesions on baseline scan in those with PET/CT consistent with TB and no previous history of TB by microbiological status.

(Left) Baseline culture positive, (Middle) TB diagnosed during follow-up, (Right) No TB diagnosed during follow-up. Infiltrates and scars are represented on a single inverted CXR as triangles, nodules as circles, with triangle size representing infiltrate/scar length and colour representing maximum FDG uptake (SUV_{max}) of each lesion. Below – Example of lesions on axial section of fused PET/CT with green star on diagram relating the represented lesion.

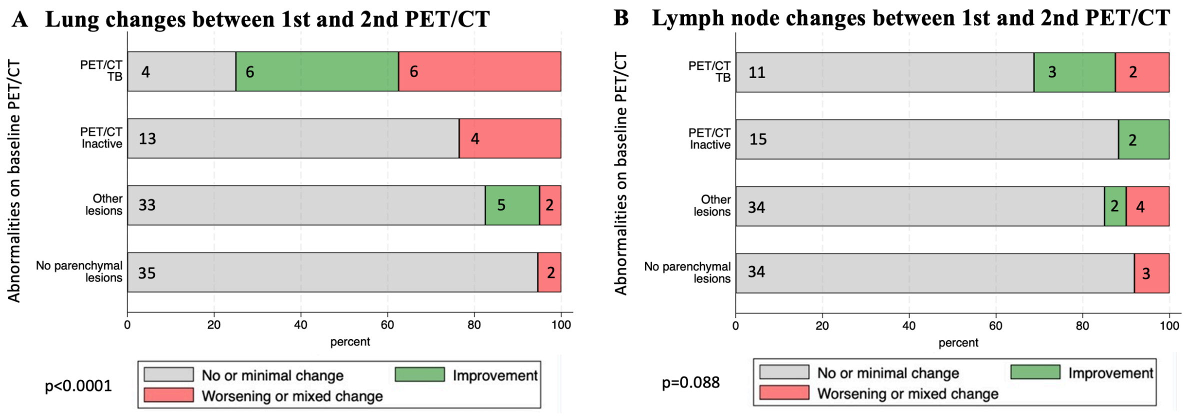

Figure S5. Lung parenchyma and lymph node changes between baseline and second PET/CT, according to baseline PET/CT lung category

Stacked bar chart showing the proportion of participants with no or minimal, improvement or worsening/mixed change between 1st and 2nd scan by baseline PET/CT classification (n=110) in (A) lung parenchymal lesions and (B) lymph node lesion. No or minimal change if there was a change in VS ≤ 1 across all lesions. Improvement if there was a reduction in VS ≥ 2 across all lesions (or clear improvement lesion with VS of 3) or resolution of a lesion. Worsening if there was an increase in VS ≥ 2 across all lesions (or clear worsening in lesion with VS of 3) or development of a new lesion. Mixed if participant had individual lesions which showed a change in VS ≤ 1 and other lesions with a change in VS ≥ 1. Compared by Fisher's exact test.

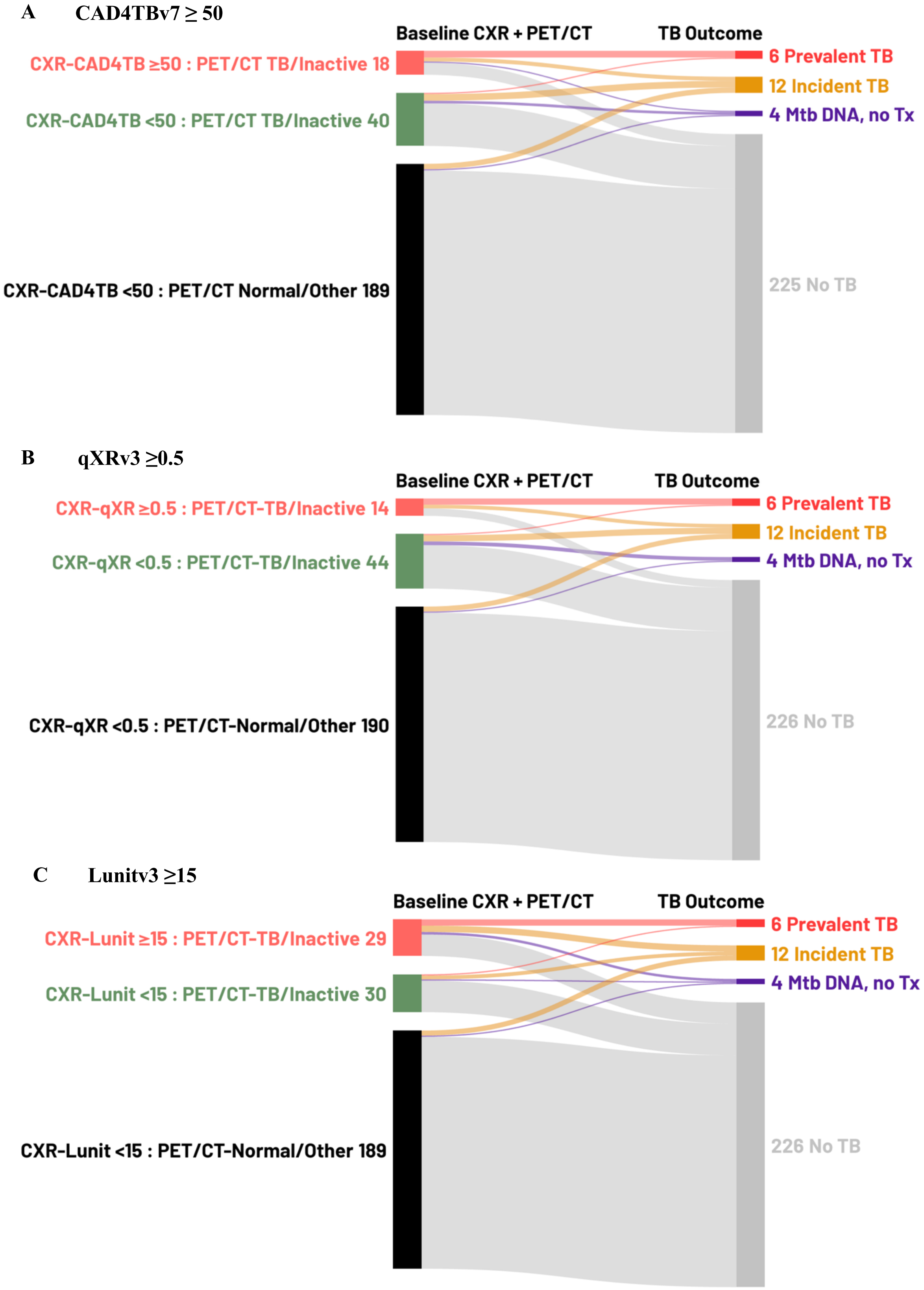

Figure S6. Sanky diagrams linking participant chest radiograph computer-aided detection (CXR-CAD) thresholds with baseline PET/CT lung category to their final TB outcome

For CXR-CAD read by (A) CAD4TB version 7.0 (CAD4TBv7), (B) qXR version 3.0.0 (qXRv3) and (C) Lunit INSIGHT CXR version 3.1.4.111 (Lunitv3). Participant numbers given for each baseline and outcome category, 247 CAD4TBv7, 248 qXRv3 and Lunitv3, 2 participants did not have a CXR, 1 no CAD4TBv7. Mtb DNA, no Tx: asymptomatic, Xpert positive, culture negative and independent clinical decision not to treat.

References

1. Boulle A, Heekes A, Tiffin N, et al. Data Centre Profile: The Provincial Health Data Centre of the Western Cape Province, South Africa. *Int J Popul Data Sci* 2019; **4**(2): 1143.
2. Esmail H, Lai RP, Lesosky M, et al. Characterization of progressive HIV-associated tuberculosis using 2-deoxy-2-[18F]fluoro-D-glucose positron emission and computed tomography. *Nat Med* 2016; **22**(10): 1090-3.
3. Medlar EM. The pathogenesis of minimal pulmonary tuberculosis; a study of 1,225 necropsies in cases of sudden and unexpected death. *Am Rev Tuberc* 1948; **58**(6): 583-611.
4. Opie EL, Aronson JD. Tubercule Bacilli in Latent Tuberculosis Lesions and in Lung Tissue without Tuberculosis Lesions. *Arch Pathol* 1927; **4**(1): 1-21.
5. Black PA, de Vos M, Louw GE, et al. Whole genome sequencing reveals genomic heterogeneity and antibiotic purification in *Mycobacterium tuberculosis* isolates. *BMC Genomics* 2015; **16**: 857.
6. Warren R, de Kock M, Engelke E, et al. Safe *Mycobacterium tuberculosis* DNA extraction method that does not compromise integrity. *J Clin Microbiol* 2006; **44**(1): 254-6.
7. Poplin R, Ruano-Rubio V, DePristo MA, et al. Scaling accurate genetic variant discovery to tens of thousands of samples. *bioRxiv* 2018: 201178. **doi:** https://doi.org/10.1101/201178
8. Munro JE, Coussens AK, Bahlo M. TBtypeR: Sensitive detection and sublineage classification of *Mycobacterium tuberculosis* complex mixed-strain infections. *Commun Biol* 2025; **8**(1): 260.
9. Napier G, Campino S, Merid Y, et al. Robust barcoding and identification of *Mycobacterium tuberculosis* lineages for epidemiological and clinical studies. *Genome Med* 2020; **12**(1): 114.
10. Thawornwattana Y, Mahasirimongkol S, Yanai H, et al. Revised nomenclature and SNP barcode for *Mycobacterium tuberculosis* lineage 2. *Microb Genom* 2021; **7**(11). 000697
11. Coscolla M, Gagneux S, Menardo F, et al. Phylogenomics of *Mycobacterium africanum* reveals a new lineage and a complex evolutionary history. *Microb Genom* 2021; **7**(2): 000477
12. Shuaib YA, Utpatel C, Kohl TA, et al. Origin and Global Expansion of *Mycobacterium tuberculosis* Complex Lineage 3. *Genes (Basel)* 2022; **13**(6).
13. Walker TM, Miotto P, Koser CU, et al. The 2021 WHO catalogue of *Mycobacterium tuberculosis* complex mutations associated with drug resistance: A genotypic analysis. *Lancet Microbe* 2022; **3**(4): e265-e73.
14. Luo W, Lin Y, Li Z, Wang W, Shi Y. Comparison of sputum induction and bronchoscopy in diagnosis of sputum smear-negative pulmonary tuberculosis: a systemic review and meta-analysis. *BMC Pulm Med* 2020; **20**(1): 146.
